# Comparative metabolomics analysis of bronchial epithelium during barrier establishment after allergen exposure

**DOI:** 10.1002/clt2.12051

**Published:** 2021-09-21

**Authors:** Juan Carlos López‐Rodríguez, Juan Rodríguez‐Coira, Sara Benedé, Coral Barbas, Domingo Barber, María Teresa Villalba, María Marta Escribese, Alma Villaseñor, Eva Batanero

**Affiliations:** ^1^ Departamento de Bioquímica y Biología Molecular Facultad de Ciencias Químicas Universidad Complutense de Madrid Madrid Spain; ^2^ Centro de Metabolómica y Bioanálisis (CEMBIO) Facultad de Farmacia Universidad San Pablo CEU CEU Universities Madrid Spain; ^3^ Instituto de Medicina Molecular Aplicada (IMMA) Departamento de Ciencias Médicas Básicas Facultad de Medicina Universidad San Pablo‐CEU CEU Universities Madrid Spain

**Keywords:** air‐liquid interface culture, bronchial epithelium, Der p 1, metabolome, respiratory allergy

## Abstract

**Background:**

Several studies have shown a correlation between an altered metabolome and respiratory allergies. The epithelial barrier hypothesis proposes that an epithelial barrier dysfunction can result in allergic diseases development. Der p 1 allergen from house dust mite is a renowned epithelial barrier disruptor and allergy initiator due to its cysteine‐protease activity. Here, we compared the metabolic profile of the bronchial epithelium exposed or not to Der p 1 during barrier establishment to understand its active role in allergy development.

**Methods:**

Calu‐3 cells were cultivated in air‐liquid interface cultures and exposed to either Der p 1 or Ole e 1 allergens during barrier establishment. The comparative metabolomics analysis of apical and basolateral media were performed using liquid chromatography and capillary electrophoresis both coupled to mass spectrometry.

**Results:**

We showed that epithelial barrier disruption by Der p 1 was associated with a specific metabolic profile, which was highly dependent on the state of the epithelium at the time of contact. Moreover, an apical‐basolateral distribution of the metabolites was also observed, indicating a compartmentalization of the response with differential metabolic patterns. A number of metabolites were changed by Der p 1, mainly related to amino acids metabolism, such as L‐arginine, L‐kynurenine and L‐methionine.

**Conclusion:**

This work is the first report on the metabolic response in human bronchial epithelial cells associated with cysteine‐protease Der p 1 activity, which could contribute to allergy development. Moreover, it supports a reformulated epithelial barrier hypothesis that might help to explain allergies and their increasing prevalence.

## 1 INTRODUCTION

During the last years, the development of inflammatory diseases such as respiratory allergies has been correlated with changes in particular metabolites, either generated by the oxidative metabolism or in response to exposome components, such as allergens.^1^, ^2^, ^3^, ^4^, ^5^ The complete analysis of metabolites (metabolomics) in a biofluid has the potential to provide information about alterations in metabolic pathways that underlie a disease.^6^, ^7^ In addition, this technology performs a rapid and accurate analysis of a wide range of low‐molecular mass molecules, which can enable to uncover new biomarkers and targets that may be applied in diagnosis, prognosis and disease treatment. To date, metabolomics based on nuclear magnetic resonance spectroscopy as well as liquid or gas chromatography coupled to mass spectrometry have been applied to numerous clinical disorders, including allergy, cancer and Alzheimer’s disease.^8^, ^9^, ^10^

Bronchial epithelial cells play a key role in the orchestration of lung immune response by secreting a wide variety of mediators, including cytokines and chemokines, among others.^11^ The function of the epithelial barrier is highly dependent on the formation of apical junctional complexes between adjacent cells, which are formed by adherens and tight junctions (TJs). Allergic diseases are characterized by a disrupted epithelial barrier.^12^ The ‘epithelial barrier hypothesis’ proposes that an epithelial barrier dysfunction can be a pivotal first‐step towards the development of allergy and others diseases.^13^ Der p 1, the main allergen of house dust mite (HDM), is a well‐recognized epithelial barrier disruptor,^14^ as well as an ‘initiator allergen’,^15^ because of its cysteine‐protease activity which can promote an allergic response to unrelated allergens. Therefore, a comparative metabolomics analysis of the bronchial epithelium exposure to Der p 1 during barrier establishment will be key to study the link between defective airway epithelial barrier and allergy development, and understand its role in this disease.

In this work, air‐liquid interface (ALI)‐cultured Calu‐3 cells were exposed to a Der p 1 or Ole e 1, an olive pollen allergen without protease activity,^16^ during barrier establishment. Comparative metabolomics analysis was performed using multiplatform analyses. To our knowledge, this is the first comparative metabolome study reported on the response of human bronchial epithelium cells to allergen exposure. Our data showed that the disruption of epithelial barrier by Der p 1 was associated with a specific metabolome profile, highly dependent on the epithelial barrier establishment at the time of exposure. In addition, an apical‐basolateral distribution of the metabolites was also detected. Finally, the metabolic pathways of particular metabolites that could be involved in the allergic response were also discussed. Overall, these data support the epithelial barrier hypothesis, which proposes that a dysfunctional barrier may underlie allergic diseases development and explain their increasing prevalence.

## 2 MATERIALS AND METHODS

### 2.1 Proteins

Natural Der p 1 (LTN‐DP1‐1, Lot. 38190) was supplied from Indoor Biotech as endotoxin‐free protein (≤ 0.03 EU/μg). Der p 1 was activated by incubation with 0.1 mM reduced glutathione (GSH; Sigma‐Aldrich) in phosphate buffered saline (PBS) at 37°C for 15 min, and its cysteine‐protease activity was checked as described.^17^ Olive pollen (IBERPOLEN SL) was used to isolate and purify Ole e 1.^16^

### 2.2 Air‐liquid interface cell cultures

Human bronchial epithelial Calu‐3 cells (ATCC No. HTB‐55, Lot. 61449062, passages 23–30) were cultured at 3 × 10^6^ cells/cm^2^ in DMEM nutrient mixture F12 (Thermo Fisher Scientific), supplemented with 2 mM L‐glutamine (Sigma‐Aldrich), 100 U/ml penicillin, 100 μg/ml streptomycin (Lonza) and 5% foetal bovine serum (FBS, Hyclone GE Healthcare), onto transwell inserts (pore size 0.4 μm, Corning 3470) at ALI, at 37°C and 5% CO_2_.

**FIGURE 1:**
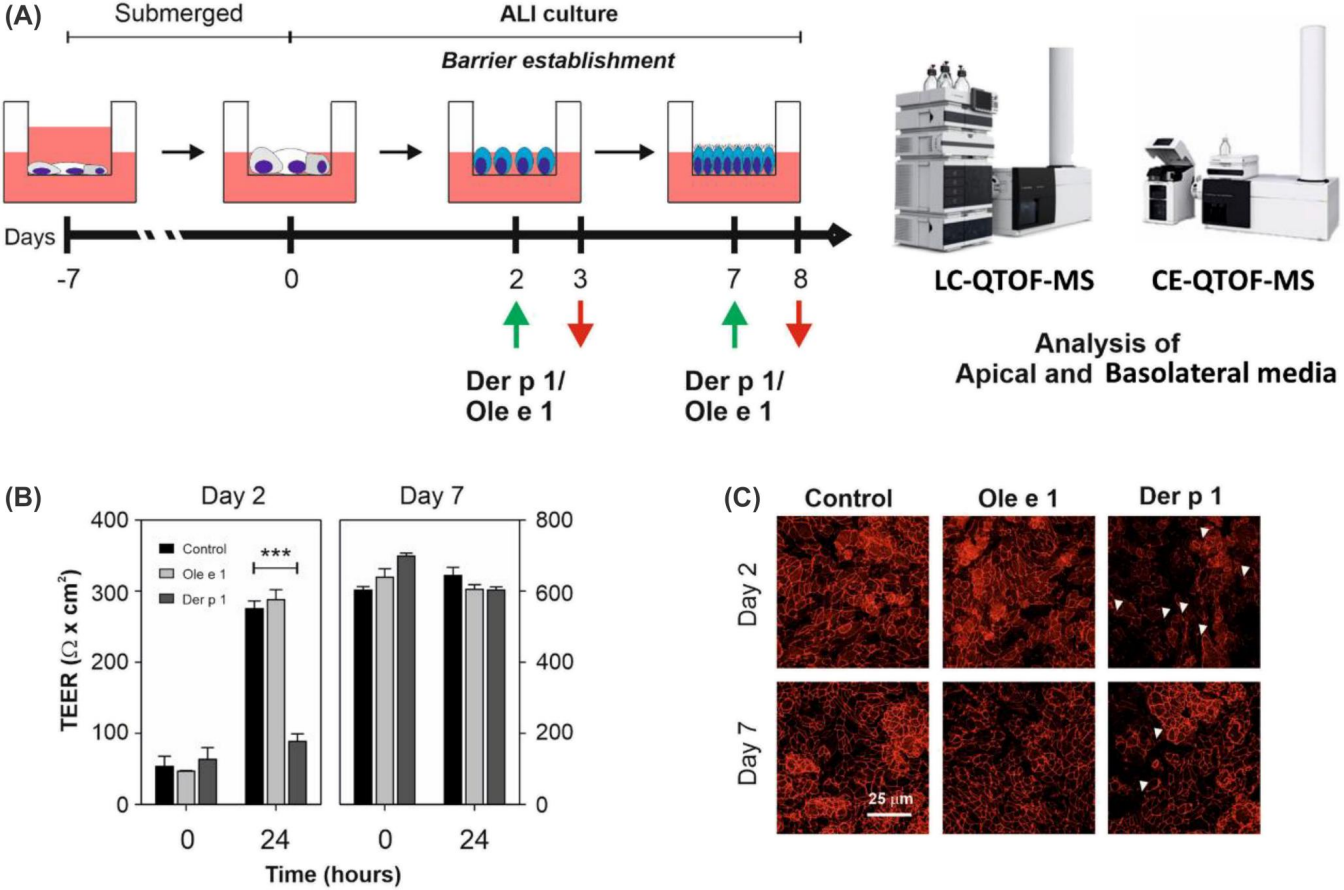
Characterization of air‐liquid interface (ALI)‐cultured Calu‐3 cells. (A) Schematic diagram of experiential protocol. Calu‐3 cells were cultured under ALI conditions and exposed to Der p 1 or Ole e 1 for 24 h during barrier establishment. Cells exposed to PBS/GSH were used as controls. Apical and basolateral media were collected, and metabolic profile was determined using liquid chromatography‐quadrupole time‐of‐flight mass spectrometry (LC‐QTOF‐MS) and capillary electrophoresis (CE)‐TOF‐MS. Green and red arrows indicate the timing of allergen addition, and the sample collection, respectively. (B) Time course of transepithelial electrical resistance values of ALI‐cultured Calu‐3 cells on the indicated time points. Data are representative of three independent experiments and are expressed as the mean ± SD of triplicate determinations. Significant differences are indicated by ***p < 0.001. (C) Confocal laser scanning microscopy analysis of zonula occludens 1 (red) expression on the indicated time points. Representative Z‐stack projection of 12–16 individual sections are shown. White arrows indicate tight junction disruptions. Scale bar = 25 μm. Control, unexposed cells; Ole e 1; cells exposed to Ole e 1; and Der p 1, cells exposed to Der p 1

ALI‐cultured cells were exposed apically to the allergen in 0.1 ml PBS containing 0.1 mM GSH on days 2 and 7: Ole e 1 (25 μg/ml) or Der p 1 (10 μg/ml). To the basolateral side, 0.5 ml of complete DMEM‐phenol free‐medium (Thermo Fisher Scientific) with 5% FBS were added. After 24 h, the apical and basolateral media were collected, and kept at −80°C until the metabolomics analysis (Figure 1A). Cells exposed only to PBS/GSH were used as control.

The apical and basolateral media were analysed using multiplatform analyses by liquid chromatography‐quadrupole time‐of‐flight mass spectrometry (LC‐QTOF‐MS, Agilent 6520 series) and capillary electrophoresis (CE)‐TOF‐MS (CE‐TOF‐MS, Agilent 6224 series) following previous workflows.^18^, ^19^, ^20^, ^21^, ^22^

The establishment of the Calu‐3 cell barrier was checked by both measuring the transepithelial electrical resistance (TEER) and immunofluorescence labelling of TJ protein zonula occludens 1 (ZO‐1). TEER was measured using an EVOM2 voltmeter device (World Precision Instruments) with an STX2 chopstick electrode, according to the manufacturer's guidelines. TEER values (Ω cm^2^) were calculated by multiplying the effective growth area (0.33 cm^2^), after subtracting the average resistance of cell‐free transwell inserts.

Detail information regarding (a) Immunofluorescence labelling and confocal laser scanning microscopy of TJs, (b) Sample preparation and Quality control preparation, (c) Metabolomics analysis, (d) Metabolite annotation, and (e) Statistical analysis are described in detail in *Supporting* *Information*. The raw data are available in Metabolights repository under accession number MTBLS1755 (http://www.ebi.ac.uk/metabolights).

## 3 RESULTS

### 3.1 *In vitro* characterization of barrier establishment by ALI‐cultured Calu‐3 cell line

TEER values and TJs between adjacent epithelial cells are accurate indicators of barrier impairment, a key orchestrator in the development of allergic diseases. Thus, Calu‐3 cells were cultured at ALI, and the establishment of the epithelial barrier was monitored by TEER measurements and TJ protein ZO‐1 staining (Figure 1). On day 2 at ALI, the epithelial barrier was still forming as indicated by the low TEER values (under 100 Ω cm^2^), and the diffuse cytoplasmic pattern of the TJ protein ZO‐1; this means that cells are non‐polarized. In contrast, the increased in both the fluorescence intensity and localization of ZO‐1 at the cell–cell contact zones, and TEER values above 500 Ω cm^2^ were indicators of the establishment of epithelial barrier by day 7 at ALI: thus, cells are polarized. Ole e 1 exposure of cells did not alter TEER values or ZO‐1 pattern, regardless of time of exposure, while exposure to Der p 1 decreased TEER values and caused a more prominent discontinuous staining pattern of ZO‐1 on day 2 than on day 7.

### 3.2 Metabolic profile of ALI‐cultured Calu‐3 cells exhibit a dual dependence on both type of exposure and cell polarity

According to the kinetic of the epithelial barrier establishment, LC‐MS+, LC‐MS− and CE‐MS were used to determine the comparative metabolic profile of ALI‐cultured Calu‐3 cells after 24 h of exposure to Ole e 1 or Der p 1 allergens, on days 2 and 7 at ALI. A total of 869 and 877 features were obtained on the apical and basolateral compartments of ALI‐cultured Calu‐3 cells, respectively, by the multiplatform analyses.

**FIGURE 2:**
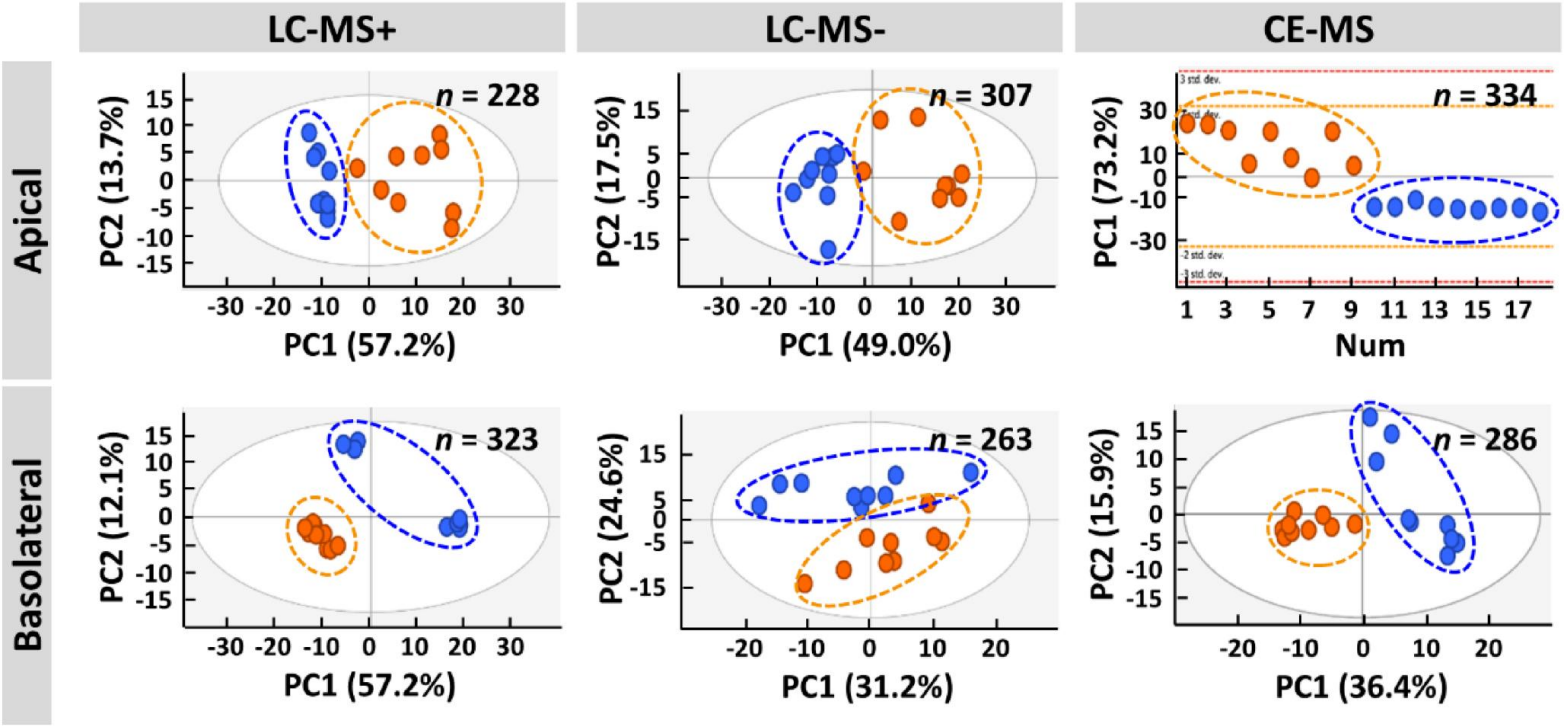
Non‐supervised PCA score plots of apical and basolateral samples from air‐liquid interface‐cultured Calu‐3 control cells and cells exposed to Der p 1 or Ole e 1 for 24 h during barrier establishment, analysed by liquid chromatography‐mass spectrometry + (LC‐MS+), LC‐MS− and capillary electrophoresis (CE)‐MS. The number (*n*) of signatures from apical and basolateral compartments are indicated. The unit variance‐scaling was applied. Three biological replicates of each group were used. Orange circle, day 2; blue circle, day 7

Non‐supervised principal component analysis (PCA) was used to detect patterns in samples according to the time of allergen‐exposure over barrier establishment on apical and basolateral compartments (Figure 2). For each platform, PCA score plots evidenced that samples were clearly separate into two clusters depending on the exposure time (day 2 vs. day 7), both on the apical and the basolateral compartments, suggesting that the metabolic response of ALI‐cultured Calu‐3 cells to an allergen strongly was dependent on the functional state of the epithelial barrier.

**FIGURE 3:**
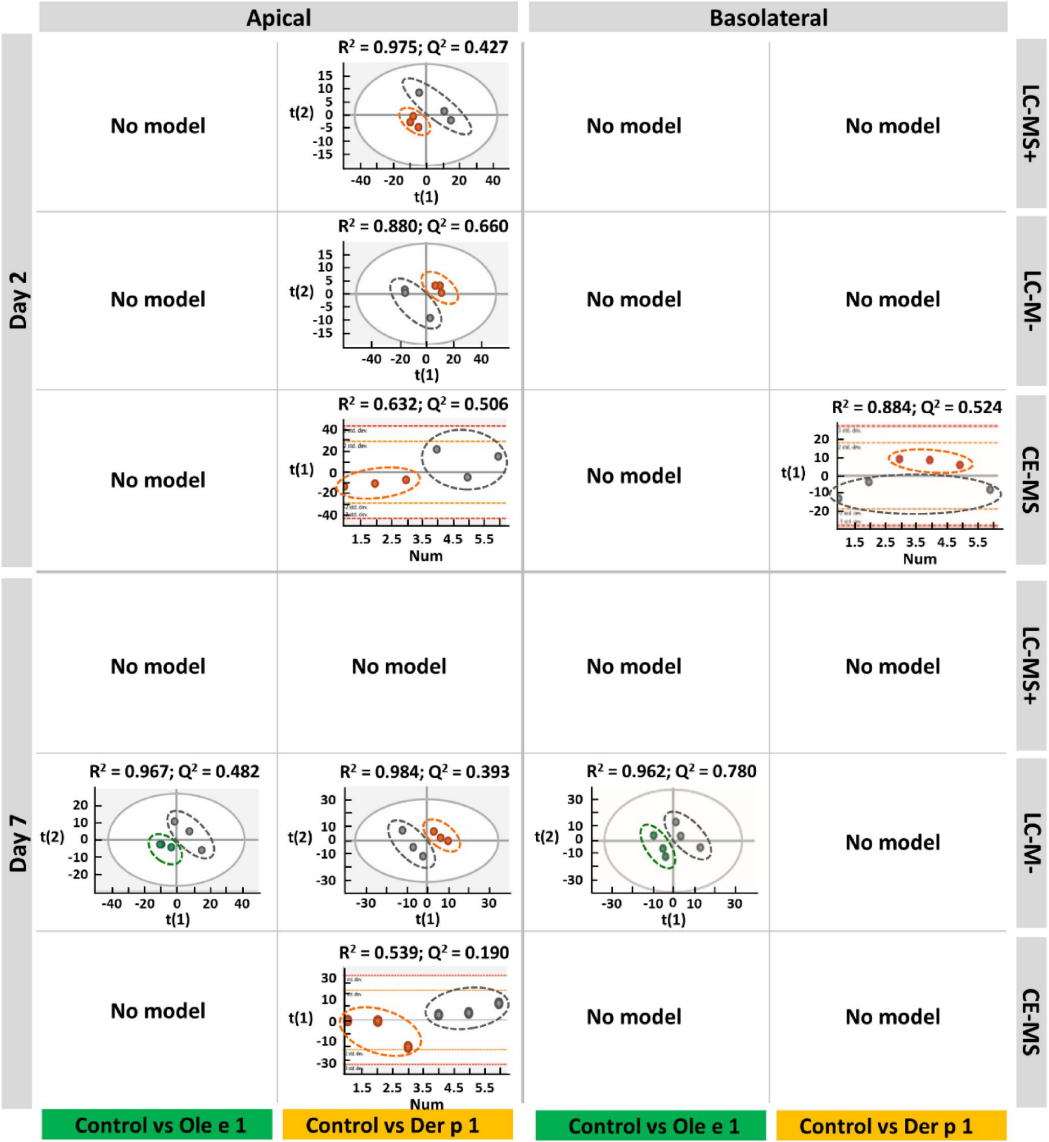
PLS‐DA models of apical and basolateral samples between control and allergen‐exposed air‐liquid interface‐cultured Calu‐3 cells on day 2 and day 7 during barrier establishment, using liquid chromatography‐mass spectrometry + (LC‐MS+), LC‐MS− and capillary electrophoresis (CE)‐MS platforms. Data were UV scaled for all the models, and *n* = 3 for all groups. Quality parameters of the models: *R* ^2^ is the variance explained by the model, and *Q* ^2^ is the predictive capability, for both 1 means 100%. ‘No model' means that not robust model was built comparing both groups

Partial least‐squares discriminant analysis (PLS‐DA) of the same samples were also performed to differentiate between control and allergen‐exposed groups, both on the apical and basolateral compartments during the time of barrier establishment (Figure 3). PLS‐DA score plots of the apical samples showed that Der p 1‐exposed group was clearly separated from the control group, regardless of the time of exposure. However, the apical metabolic profiles of Ole e 1‐exposed and control groups did no exhibit significant differences, as only one PLS‐DA model was obtained on day 7 by LC‐MS− platform. The differences observed between Ole e 1 and Der p 1 can be explain by the effect of the latter on the epithelial barrier: Der p 1, but no Ole e 1, altered TEER values and ZO‐1 expression pattern. The *R* ^2^ values (variance explained) for all models were 0.984–0.539, and their *Q* ^2^ (predictive capability) were 0.660–0.190. Samples analysed by LC‐M− showed higher and most robust PLS‐DA models. Although some *Q* ^2^ values were low, the separation between groups observed in the obtained PLS‐DA models suggest that ALI‐cultured Calu‐3 cells exposed to Der p 1 exhibited a distinct pattern in the metabolic profile on the apical compartment. Nevertheless, for most of the basolateral dataset, no PLS‐DA models were obtained, indicating that there were no notable significant differences between groups with the type of exposure at different times of barrier establishment (Figure 3). Only one model was built for Der p 1‐exposed group versus control on 2 days using CE‐MS platform, and this suggest that the differences in metabolic profile between both groups could be mainly due to the amino acids as this technique allows their detection. And, a single PLS‐DA model was obtained for Ole e 1 versus control on day 7 using LC‐MS−. On the basis of these data, the apical and basolateral compartments exhibited different metabolic profiles, mainly, in Der p 1‐exposed cells.

### 3.3 Annotation and identification of metabolites released by ALI‐cultured Calu‐3 cells after allergen exposure during barrier establishment

Univariate analysis was performed to define which metabolites were differentially released among the three groups. A total of 292 and 196 differential features were obtained among all the experimental groups on the apical compartment on days 2 and 7, respectively. For the basolateral compartment, 118 and 60 differential features were detected on days 2 and 7, respectively. The number of features were significant higher on the apical compartment than on the basolateral one in all groups.

**FIGURE 4:**
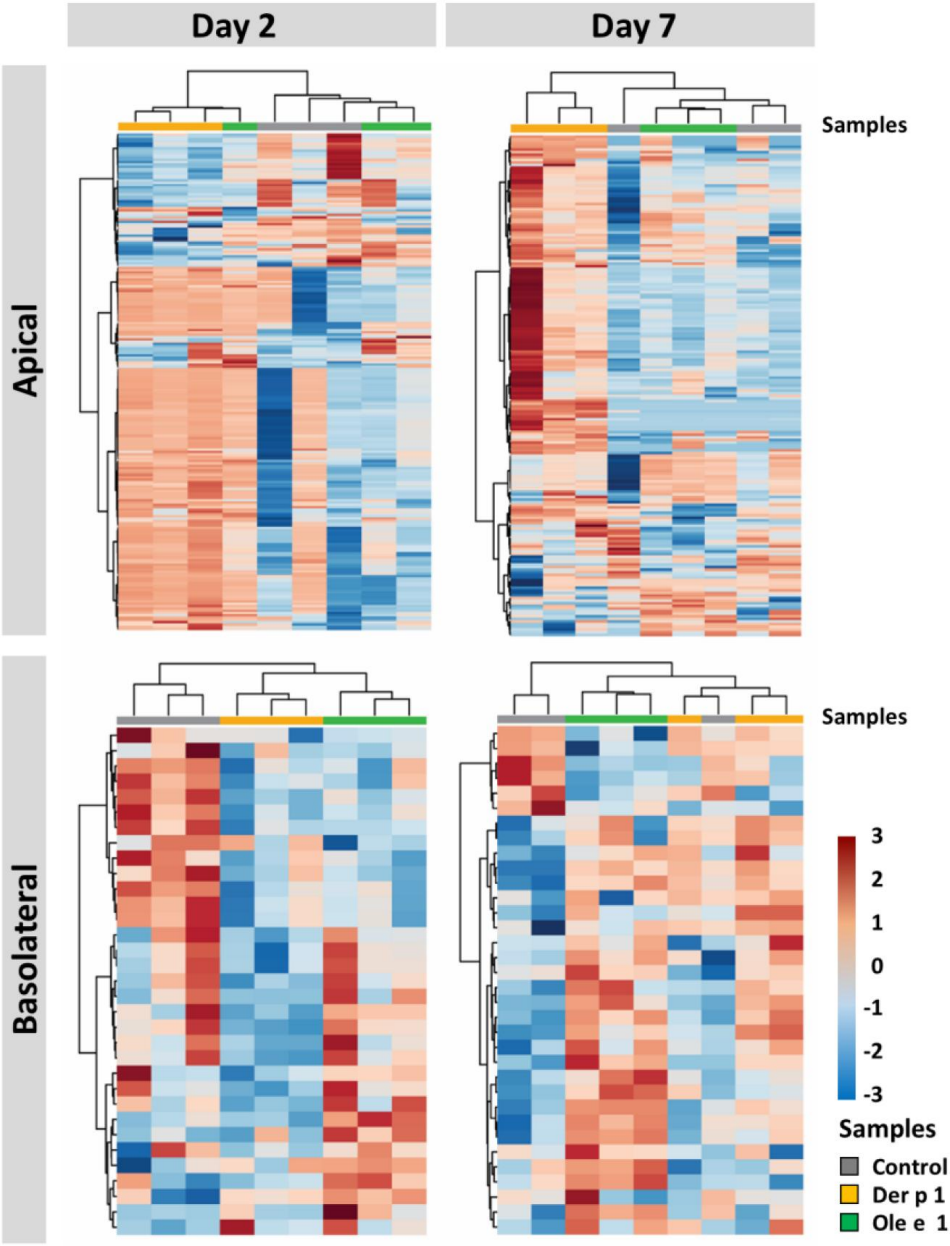
Heatmaps of significant metabolites from air‐liquid interface‐cultured Calu‐3 cells exposed to Der p 1 or Ole e 1 allergens in comparison to control cells, on days 2 and 7 during barrier establishment. Significantly differed metabolites were selected using Mann‐Whitney *U* test (*p* < 0.05), and hierarchical clustered using Ward's algorithm, with dendrograms to represent the distance between samples: apical data (*n* = 18, metabolites *n* = 409); and basolateral data (*n* = 18, metabolites *n* = 69). Colours represent the levels of metabolites (rows) from the biological samples (columns): red, high levels and blue, low levels

**FIGURE 5:**
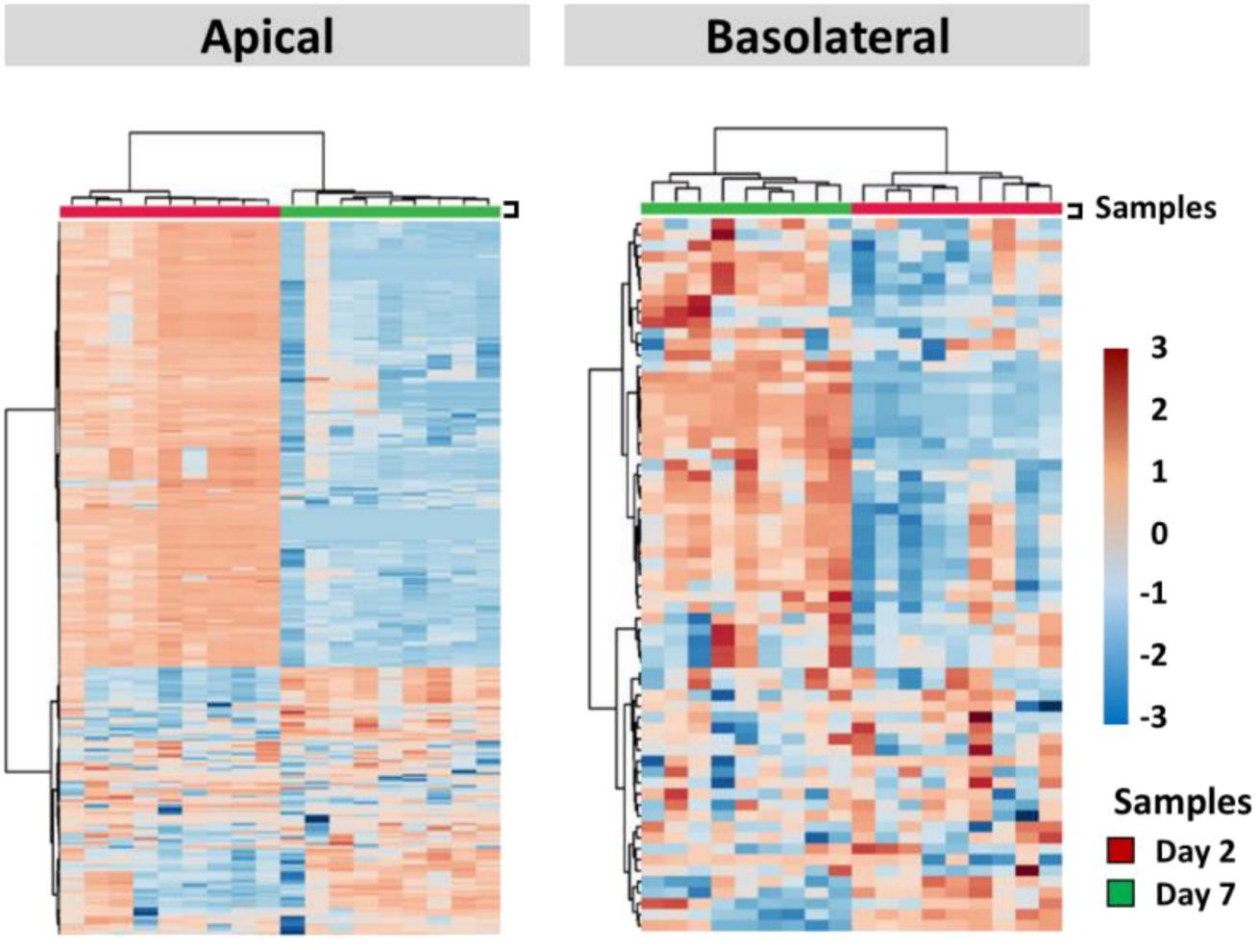
Heatmaps of significant metabolites from air‐liquid interface‐cultured Calu‐3 cells on day 2 versus day 7 during barrier establishment. Significantly differed metabolites (*p* < 0.05) with a false discovery rate ≤0.1 obtained using liquid chromatography‐mass spectrometry + (LC‐MS+), LC‐MS− and capillary electrophoresis‐MS platforms were selected: apical data (*n* = 18, metabolites *n* = 338); and basolateral data (*n* = 18, metabolites *n* = 65). Colours represent the levels of metabolites (rows) from the biological samples (columns): red, high levels; and blue, low levels

Heatmaps were generated to visualize the hierarchical cluster analysis of metabolic profiles from the three groups (Figure 4). These representations indicate similarity/dissimilarity (as distance measure) among groups. Each square in the heatmap dendrogram represents the abundance level of a single metabolite in a single sample. It was observed again that the metabolome of cells exposed to Der p 1 could be clearly separated, both from control and Ole e 1‐exposed cells within the time of barrier establishment, suggesting a particular metabolome for the cells after exposure to this allergen (Figure 4). The metabolome of the three groups could clearly discriminate from each other whatever the time of barrier establishment. It is worth noting that the number of decreased metabolites was notably higher on the basolateral compartments than on the apical one of Der p 1‐exposed cells on day 2 compared to the other groups. Finally, Der p 1 and Ole e 1 were linked together by the dendrogram suggesting that the metabolic profile of both allergens share similitudes on the basolateral compartment. In addition, heatmaps showed that samples could be clearly separated with the time of barrier establishment both on the apical and the basolateral compartments (Figure 5). Interestingly, metabolites that were increased on day 7, tended to be decreased on day 2, and vice versa (being more evident on the apical compartment). Significant features were annotated, as illustrated in Tables S1 and S2, amino acids such as L‐alanine, L‐isoleucine or L‐serine were increased between 7 and 10 fold on the apical compartment of Der p 1 exposed cells on day 7 compared to day 2.

After annotation, a total of 67 and 20 extracellular metabolites were obtained for the apical and basolateral compartments, respectively (Table S1–S4). These metabolites encompass biochemical subclasses such as: amino acids, peptides and analogues (i.e., L‐alanine, asparaginylalanine or pipecolic acid); carbohydrates and carbohydrate conjugates (i.e., D‐fructose or fructosyl‐lysine); diacyl glycerophospholipids (i.e., PI(37:7) or PS(41:4)); fatty acids and conjugates (i.e., mevalonic acid); pterins and derivatives (i.e., folinic acid); carbonyl compounds (L‐kynurenine); and pyridinecarboxylic acids (i.e., niacinamide), among others. Amino acids, peptides and analogues were the main groups of metabolites that were significantly altered in both cellular compartments after allergen exposure, both on days 2 and 7. On day 2, L‐glutamic, L‐isoleucine, L‐kynurenine, L‐methionine, L‐tyrosine, L‐tryptophan and L‐valine were among the top 10 metabolites exhibiting the highest levels in the apical compartment after Der p 1‐exposure; while on day 7, were L‐alanine, L‐arginine, L‐glycine, L‐glutamine, L‐isoleucine and L‐methionine, among others. Interestingly, metabolites related to lipid metabolism, including sphingolipids, glycerophospholipids, inositol phosphates and fatty acid thioesters were decreased after Der p 1‐exposure, both on days 2 and 7. Analysis of metabolic pathways altered in ALI‐cultured Calu‐3 cells after allergen exposure was performed using IMPaLA (Table S5). Pathway analysis showed that most of the significantly changed pathways after allergen exposure in comparison to control cells were related to amino acid metabolism and transport, including arginine metabolism, glycine, serine and threonine metabolism, phenylalanine and tyrosine metabolism and tryptophan metabolism.

## 4 DISCUSSION

During the last years, metabolomics studies have revealed the alteration of relevant biological pathways in relation to diseases, including asthma and other allergic diseases.^1^, ^2^, ^3^, ^4^, ^5^, ^6^, ^7^, ^8^ In this work, the comparative metabolic profile of ALI‐cultured Calu‐3 cells was obtained after the exposure to Der p 1 or Ole e 1 allergens for 24 h, on days 2 and 7 of culture. The model at day 2 mimics an immature/impaired airway epithelium in early childhood with viral infections,^23^ while the model at day 7 represents a mature epithelium of a healthy individual. Overall, our data show the strong dependence of the metabolic response to allergen exposure on the epithelium state at the time of contact, as indicated by the differences detected between metabolic profiles on day 2 and 7 of culture at ALI. These data agreed with the functional state‐dependence described for the disruption of the airway barrier by Der p 1, mainly affecting an immature/impaired epithelium (day 2), but not on a healthy one (day 7).^24^ Similarly, a differentiation state‐dependent of cytokine release by ALI‐cultured NHBE cells in response to Ole e 1 has also been described.^25^ Moreover, the multivariate analysis of the samples revealed further differences between the epithelial cell response to Ole e 1 and Der p 1 allergens. Exposure to Der p 1 significantly altered the metabolic profile of the cells but no significant differences were detected between metabolic profiles of Ole e 1‐exposed cells compared to control cells. These differences could be partly explained by the cysteine‐protease activity of Der p 1 that previous studies have shown to contribute to the allergic response by different mechanisms.^14^ In this sense, Der p 1 disrupts bronchial epithelial barrier by cleavage of the TJ proteins (occludins, claudins and ZO‐1), which results in cell signalling alteration. Here, we showed that disruption of the epithelial barrier by Der p 1 was associated with a specific metabolic profile dependent on the epithelial barrier establishment at the time of exposure (day 2 vs. day 7). In addition, the data also demonstrated differences in the apical versus basolateral distribution of metabolites, suggesting that independent signalling pathways may occur in the apical and basolateral compartments of the polarized bronchial epithelial after allergen exposure. Epithelial cell polarity results from the cell barrier formation, which depends on TJs and adherens junctions established between neighbouring cells. Cell polarity is crucial in maintenance of lung homoeostasis. Therefore, the disruption of cell polarity by environmental insults such as proteases, could have an important function in the development of allergy.

Several pathways were altered after allergen exposure during epithelial barrier establishment. Marked changes in amino acid metabolism were found. High levels of the amino acids glycine, serine, and threonine, which shares biochemical pathways, were detected on the apical compartment of ALI‐cultured Calu‐3 cells after Der p 1 exposure on day 7. The metabolic pathways of these amino acids have been associated with severe asthma in children.^26^ Serine is a precursor of the amino acids glycine and cysteine that are components of the tripeptide GSH, which plays a key role in the regulation of lung pro‐inflammatory response. Similarly, high levels of glutamine were also found on the apical compartment of this culture. The observed increased levels of the essential GSH precursor suggests that GSH metabolism and synthesis are altered in Der p 1, exposed cells on day 7. In addition, serine is also a precursor of sphingolipids and glycerophospholipids, which are decreased after Der p 1 exposure during barrier establishment.

Moreover, high levels of the branched chain amino acids L‐valine and L‐isoleucine were also detected on the apical compartment of ALI‐cultured Calu‐3 cells after Der p 1 exposure along the culture. It has been reported that branched amino acid supplementation modulate the immune response by promoting cell proliferation and regulation of cytokine secretion.^27^ This was accompanied by high levels of L‐histidine and L‐methionine on the apical compartment of ALI‐cultured Calu‐3 cells after Der p 1 exposure. Histidine is the precursor of histamine, an inflammatory mediator and disruptor of the epithelial barrier,^28^ whose levels are elevated in allergic patients. Methionine plays an important role in the synthesis of proteins involved in the immune response. This has been identified as a biomarker of asthma, along with glutamine and histidine,^29^ and all three altered at seizure stage of pollinic patients.^30^

On the other hand, L‐tryptophan, L‐arginine and L‐kynurenine were significantly increased on the apical compartment of ALI‐cultured Calu‐3 cells after Der p 1 exposure but not on the basolateral one, compared to both, control and Ole e 1‐exposed cells. These metabolites have been associated to allergy.^31^, ^32^ The essential amino acid L‐tryptophan is breakdown by the indoleamine 2,3‐dioxygenase (IDO) enzyme in N‐formyl‐kynurenine via the kynurenine pathway, which also include the metabolite kynurenine.^33^ IDO‐1 is the predominant isoform expressed in the lung by epithelial cells among others.^34^ This enzyme has been involved in allergic inflammation by controlling local levels of tryptophan.^35^, ^36^, ^37^, ^38^ Defective IDO activity has been reported in rhinitis allergic patient and in airway epithelial primary cultures and cell lines after HDM exposure.^39^, ^40^ Moreover, high tryptophan levels have been described in serum of pollinic patients^35^ and linked to unresponsiveness to immunotherapy in different animal models.^41^, ^42^, ^43^, ^44^ We found a decreased kynurenine/tryptophan ratio on the apical compartment of ALI‐cultured cells exposed to Der p 1 on day 2, when the barrier is not yet established. Interestingly, this ratio was not altered on day 7, when the barrier is established. It has been shown that kynurenine contributes to tolerance induction against allergens and has been associated with low activity of IDO.^41^, ^45^, ^46^ Overall, our data support the notion that exposure to Der p 1 induces an inflammatory response on the apical compartment of ALI‐cultured Calu‐3 cells on day 2. L‐arginine plays a key role in the regulation of airway function via the production of the bronchodilating nitric oxide (NO) by the nitric oxide synthase (NOS). Both arginine and NO have been extensively reported to be increased in asthma.^47^, ^48^, ^49^ However, controversy exits about their role on airway responsiveness and remodelling.^50^ Thus, an increased NOS activity has been associated to a pro‐inflammatory state, whereas an up‐regulation of arginase activity has been linked to an anti‐inflammatory state and inhibition of Th2 response.^50^, ^51^, ^52^, ^53^ Moreover, increased amounts of intracellular NO have been shown to inhibit the mitochondrial respiratory chain, leading cells to rely on Warburg metabolism for energy generation.^54^ Interestingly, asymmetrical dimethyl arginine (ADMA), another important metabolite in arginine metabolism, was increased on the apical compartment after Der p 1 exposure only on day 7. ADMA is a potent NOS inhibitor, and low levels of this metabolite have been associated with decreased airway function in asthmatic patients.^55^, ^56^

The lung is a metabolically highly active organ whose glucose consumption exceeds that of other organs.^57^ Our data suggested that ALI‐cultured Calu‐3 cells shift their metabolism to aerobic glycolysis or Warburg metabolism for ATP production after exposure to Der p 1 on day 2, as indicated by the accumulation of pantothenic acid on the apical compartment. The pantothenic acid is precursor of the synthesis of coenzyme A, which is essential in the metabolism of fatty acids and the citric acid cycle. The low levels of pyridoxal found on the basolateral media after Der p 1 treatment supports the notion that the epithelial cells are using aerobic glycolysis as pyridoxal liberates glucose monomers form glycogen (glycogenolysis).^54^ Warburg metabolism is switched on in response to cellular activation in numerous cell types and in highly proliferative cells, such as T cells and macrophages.^58^, ^59^ However, further studies are needed to understand its role in the immature epithelial barrier.

To our current knowledge, this is the first time a study has reported on the metabolic response of human bronchial epithelial cells to allergen exposure. Together, our data proved that the disruption of epithelial barrier by the cysteine‐protease Der p 1 activity was associated with a specific metabolic profile, which could contribute to allergy development. Moreover, this metabolic response was highly dependent on the epithelial barrier establishment at the time of allergen exposure. The metabolic changes may provide a key to uncovering the role of the epithelial barrier in the development of allergic diseases at the molecular level. Finally, these data support a reformulated epithelial barrier hypothesis, which postulates that a dysfunctional barrier can underlie allergic diseases development and explain their increasing prevalence.

## AUTHOR CONTRIBUTIONS

Conceptualization: Eva Batanero; Methodology: Juan Carlos López‐Rodríguez, Sara Benedé and Eva Batanero (cellular experiments), and Juan Rodríguez‐Coira and Alma Villaseñor (metabolic experiments); Data analysis: Juan Carlos López‐Rodríguez, Juan Rodríguez‐Coira, Alma Villaseñor and Eva Batanero; Writing, original draft: Juan Carlos López‐Rodríguez, Juan Rodríguez‐Coira, Alma Villaseñor and Eva Batanero; Writing, review and editing: Juan Carlos López‐Rodríguez, Juan Rodríguez‐Coira, Sara Benedé, Coral Barbas, Alma Villaseñor, Domingo Barber, María Teresa Villalba, María Marta Escribese, and Eva Batanero; Supervision: Coral Barbas and Eva Batanero; Funding acquisition: María Marta Escribese, Domingo Barber and María Teresa Villalba.

## PREVIOUS PRESENTATIONS

Some data from this study have been presented at the 2019 European Academy of Allergy and Clinical Immunology (EAACI) congress in Lisbon (June 1–5), with an abstract number OA0080.

## Supporting information

Supporting Information 1

## References

[1] Kelly RS , Dahlin A , McGeachie MJ , et al. Asthma metabolomics and the potential for integrative omics in research and the clinic. Chest. 2017;151:262‐277. 27776981 10.1016/j.chest.2016.10.008 PMC5310123

[2] Villaseñor A , Rosace D , Obeso D , et al. Allergic asthma: an overview of metabolomic strategies leading to the identification of biomarkers in the field. Clin Exp Allergy. 2017;47:442‐456. 28160515 10.1111/cea.12902

[3] Barber D , Villaseñor A , Escribese MM . Metabolomics strategies to discover new biomarkers associated to severe allergic phenotypes. Asia Pac Allergy. 2019;9:e37. 31720248 10.5415/apallergy.2019.9.e37 PMC6826109

[4] Pite H , Aguiar L , Morello J , et al. Metabolic dysfunction and asthma: current perspectives. J Asthma Allergy. 2020;13:237‐247. 32801785 10.2147/JAA.S208823 PMC7394599

[5] Spertini F . Metabolomics and allergy: opening pandora's box. J Allergy Clin Immunol. 2020;145:782‐784. 31981625 10.1016/j.jaci.2020.01.012

[6] Dias DA , Koal T . Progress in metabolomics standardisation and its significance in future clinical laboratory medicine. EJIFCC. 2016;27:331‐343. 28149265 PMC5282916

[7] Evans ED , Duvallet C , Chu ND , et al. Predicting human health from biofluid‐based metabolomics using machine learning. Sci Rep. 2020;10:17635. 33077825 10.1038/s41598-020-74823-1 PMC7572502

[8] Obeso D , Mera‐Berriatua L , Rodríguez‐Coira J , et al. Multi‐omics analysis points to altered platelet functions in severe food‐associated respiratory allergy. Allergy. 2018;73:2137‐2149. 30028518 10.1111/all.13563

[9] Zetterberg H , Burnham SC . Blood‐based molecular biomarkers for Alzheimer's disease. Mol Brain. 2019;12:26. 30922367 10.1186/s13041-019-0448-1 PMC6437931

[10] Kowalczyk T , Ciborowski M , Kisluk J , Kretowski A , Barbas C . Mass spectrometry based proteomics and metabolomics in personalized oncology. Biochim Biophys Acta (BBA) – Mol Basis Dis. 2020;1866:165690. 10.1016/j.bbadis.2020.165690 31962175

[11] López‐Rodríguez JC , Benedé S , Barderas R , Villalba M , Batanero E . Airway epithelium plays a leading role in the complex framework underlying respiratory allergy. J Investig Allergol Clin Immunol. 2017;27:346‐355. 10.18176/jiaci.0201 29199961

[12] Hellings PW , Steelant B . Epithelial barriers in allergy and asthma. J Allergy Clin Immunol. 2020;145:1499‐1509. 32507228 10.1016/j.jaci.2020.04.010 PMC7270816

[13] Akdis CA . Does the epithelial barrier hypothesis explain the increase in allergy, autoimmunity and other chronic conditions? Nat Rev Immunol. Published online April 12, 2021. 10.1038/s41577-021-00538-7 33846604

[14] Chevigné A , Jacquet A . Emerging roles of the protease allergen Der p 1 in house dust mite‐induced airway inflammation. J Allergy Clin Immunol. 2018;142:398‐400. 29906529 10.1016/j.jaci.2018.05.027

[15] Zhang J , Chen J , Robinson C . Cellular and molecular events in the airway epithelium defining the interaction between house dust mite group 1 allergens and innate defences. Int J Mol Sci. 2018;19. 10.3390/ijms19113549 PMC6274810 30423826

[16] Villalba M , Batanero E , López‐Otín C , et al. The amino acid sequence of Ole e I, the major allergen from olive tree (*Olea europaea*) pollen. Eur J Biochem. 1993;216:863‐869. 8404906 10.1111/j.1432-1033.1993.tb18208.x

[17] Schulz O , Sewell HF , Shakib F . A sensitive fluorescent assay for measuring the cysteine protease activity of Der p 1, a major allergen from the dust mite Dermatophagoides pteronyssinus. Mol Pathol. 1998;51:222‐224. 9893750 10.1136/mp.51.4.222 PMC395641

[18] Ciborowski M , Javier Rupérez F , Martínez‐Alcázar MP , et al. Metabolomic approach with LC‐MS reveals significant effect of pressure on diver's plasma. J Proteome Res. 2010;9:4131‐4137. 20504017 10.1021/pr100331j

[19] Canuto GAB , Castilho‐Martins EA , Tavares M , López‐Gonzálvez A , Rivas L , Barbas C . CE‐ESI‐MS metabolic fingerprinting of Leishmania resistance to antimony treatment. Electrophoresis. 2012;33:1901‐1910. 22740478 10.1002/elps.201200007

[20] Gil‐de‐la‐Fuente A , Godzien J , Saugar S , et al. CEU mass mediator 3.0: a metabolite annotation tool. J Proteome Res. 2019;18:797‐802. 30574788 10.1021/acs.jproteome.8b00720

[21] González‐Riano C , Dudzik D , Garcia A , et al. Recent developments along the analytical process for metabolomics workflows. Anal Chem. 2020;92:203‐226. 31625723 10.1021/acs.analchem.9b04553

[22] Mamani‐Huanca M , Gradillas A , Gil de la Fuente A , López‐Gonzálvez Á , Barbas C . Unveiling the fragmentation mechanisms of modified amino acids as the key for their targeted identification. Anal Chem. 2020;92:4848‐4857. 32119527 10.1021/acs.analchem.9b04313

[23] Holt PG , Sly PD . Prevention of allergic respiratory disease in infants: current aspects and future perspectives. Curr Opin Allergy Clin Immunol. 2007;7:547‐555. 17989533 10.1097/ACI.0b013e3282f14a17

[24] López‐Rodríguez JC , González M , Bogas G , Mayorga C , Villalba M , Batanero E . Epithelial permeability to ole e 1 is more dependent on functional bronchial epithelium‐state than on Der P 1‐Protease activity that acts as adjuvant to the bystander allergen. J Investig Allergol Clin Immunol. Published online October 8, 2020. 10.18176/jiaci.0603 33030433

[25] López‐Rodríguez JC , Solís‐Fernández G , Barderas R , Villalba M , Batanero E . Effects of Ole e 1 on human bronchial epithelial cells cultured at the air‐liquid interface. J Investig Allergol Clin Immunol. 2018;28:186‐189. 10.18176/jiaci.0227 29939136

[26] Fitzpatrick AM , Park Y , Brown LAS , Jones DP . Children with severe asthma have unique oxidative stress‐associated metabolomic profiles. J Allergy Clin Immunol. 2014;133:258‐261.e1‐8. 24369802 10.1016/j.jaci.2013.10.012 PMC3915866

[27] Negro M , Giardina S , Marzani B , Marzatico F . Branched‐chain amino acid supplementation does not enhance athletic performance but affects muscle recovery and the immune system. J Sports Med Phys Fit. 2008;48:347‐351. 18974721

[28] Steelant B , Seys SF , Van Gerven L , et al. Histamine and T helper cytokine‐driven epithelial barrier dysfunction in allergic rhinitis. J Allergy Clin Immunol. 2018;141:951‐963.e8. 29074456 10.1016/j.jaci.2017.08.039

[29] Jung J , Kim S‐H , Lee H‐S , et al. Serum metabolomics reveals pathways and biomarkers associated with asthma pathogenesis. Clin Exp Allergy. 2013;43:425‐433. 23517038 10.1111/cea.12089

[30] Zhou Y‐J , Li L‐S , Sun J‐L , Guan K , Wei J‐F . 1H NMR‐based metabolomic study of metabolic profiling for pollinosis. World Allergy Organ J. 2019;12:100005. 30937130 10.1016/j.waojou.2018.11.005 PMC6439407

[31] Gostner JM , Becker K , Kofler H , Strasser B , Fuchs D . Tryptophan metabolism in allergic disorders. Int Arch Allergy Immunol. 2016;169:203‐215. 27161289 10.1159/000445500 PMC5433561

[32] Magnus MC , Karlstad Ø , Midtun Ø , et al. Maternal plasma total neopterin and kynurenine/tryptophan levels during pregnancy in relation to asthma development in the offspring. J Allergy Clin Immunol. 2016;138:1319‐1325. 27221136 10.1016/j.jaci.2016.02.032 PMC5073035

[33] Van der Leek AP , Yanishevsky Y , Kozyrskyj AL . The kynurenine pathway as a novel link between allergy and the gut microbiome. Front Immunol. 2017;8:1374. 29163472 10.3389/fimmu.2017.01374 PMC5681735

[34] Jia S , Guo P , Ge X , Wu H , Lu J , Fan X . Overexpression of indoleamine 2, 3‐dioxygenase contributes to the repair of human airway epithelial cells inhibited by dexamethasone via affecting the MAPK/ERK signaling pathway. Exp Ther Med. 2018;16:282‐290. 29896251 10.3892/etm.2018.6163 PMC5995046

[35] Kositz C , Schroecksnadel K , Grander G , Schennach H , Kofler H , Fuchs D . High serum tryptophan concentration in pollinosis patients is associated with unresponsiveness to pollen extract therapy. Int Arch Allergy Immunol. 2008;147:35‐40. 18446051 10.1159/000128584

[36] Raitala A , Karjalainen J , Oja SS , Kosunen TU , Hurme M . Indoleamine 2,3‐dioxygenase (IDO) activity is lower in atopic than in non‐atopic individuals and is enhanced by environmental factors protecting from atopy. Mol Immunol. 2006;43:1054‐1056. 15992929 10.1016/j.molimm.2005.06.022

[37] Buyuktiryaki B , Sahiner UM , Girgin G , et al. Low indoleamine 2,3‐dioxygenase activity in persistent food allergy in children. Allergy. 2016;71:258‐266. 26449488 10.1111/all.12785

[38] Wu H , Gong J , Liu Y . Indoleamine 2, 3‐dioxygenase regulation of immune response (Review). Mol Med Rep. 2018;17:4867‐4873. 29393500 10.3892/mmr.2018.8537

[39] Ciprandi G , De Amici M , Tosca M , Fuchs D . Tryptophan metabolism in allergic rhinitis: the effect of pollen allergen exposure. Hum Immunol. 2010;71:911‐915. 20540982 10.1016/j.humimm.2010.05.017

[40] Aldajani WA , Salazar F , Sewell HF , Knox A , Ghaemmaghami AM . Expression and regulation of immune‐modulatory enzyme indoleamine 2,3‐dioxygenase (IDO) by human airway epithelial cells and its effect on T cell activation. Oncotarget. 2016;7:57606‐57617. 27613847 10.18632/oncotarget.11586 PMC5295376

[41] Taher YA , Piavaux BJA , Gras R , et al. Indoleamine 2,3‐dioxygenase‐dependent tryptophan metabolites contribute to tolerance induction during allergen immunotherapy in a mouse model. J Allergy Clin Immunol. 2008;121:983‐991. 18179817 10.1016/j.jaci.2007.11.021

[42] Ito H , Hoshi M , Ohtaki H , et al. Ability of IDO to attenuate liver injury in alpha‐galactosylceramide‐induced hepatitis model. J Immunol. 2010;185:4554‐4560. 20844202 10.4049/jimmunol.0904173

[43] Sucher R , Fischler K , Oberhuber R , et al. IDO and regulatory T cell support are critical for cytotoxic T lymphocyte‐associated Ag‐4 Ig‐mediated long‐term solid organ allograft survival. J Immunol. 2012;188:37‐46. 22131334 10.4049/jimmunol.1002777 PMC3249458

[44] Sundaram G , Lim CK , Brew BJ , Guillemin GJ . Kynurenine pathway modulation reverses the experimental autoimmune encephalomyelitis mouse disease progression. J Neuroinflammation. 2020;17:176. 32505212 10.1186/s12974-020-01844-y PMC7276083

[45] von Bubnoff D , Bieber T . The indoleamine 2,3‐dioxygenase (IDO) pathway controls allergy. Allergy. 2012;67:718‐725. 22519427 10.1111/j.1398-9995.2012.02830.x

[46] Campesato LF , Budhu S , Tchaicha J , et al. Blockade of the AHR restricts a Treg‐macrophage suppressive axis induced by L‐Kynurenine. Nat Commun. 2020;11:4011. 32782249 10.1038/s41467-020-17750-z PMC7419300

[47] Maarsingh H , Zuidhof AB , Bos IST , et al. Arginase inhibition protects against allergen‐induced airway obstruction, hyperresponsiveness, and inflammation. Am J Respir Crit Care Med. 2008;178:565‐573. 18583571 10.1164/rccm.200710-1588OC

[48] Ghosh S , Janocha AJ , Aronica MA , et al. Nitrotyrosine proteome survey in asthma identifies oxidative mechanism of catalase inactivation. J Immunol. 2006;176:5587‐5597. 16622028 10.4049/jimmunol.176.9.5587

[49] Lara A , Khatri SB , Wang Z , et al. Alterations of the arginine metabolome in asthma. Am J Respir Crit Care Med. 2008;178:673‐681. 18635886 10.1164/rccm.200710-1542OC PMC2556449

[50] Mabalirajan U , Ahmad T , Leishangthem GD , et al. Beneficial effects of high dose of L‐arginine on airway hyperresponsiveness and airway inflammation in a murine model of asthma. J Allergy Clin Immunol. 2010;125:626‐635. 20153031 10.1016/j.jaci.2009.10.065

[51] Morris CR , Poljakovic M , Lavrisha L , Machado L , Kuypers FA , Morris SM . Decreased arginine bioavailability and increased serum arginase activity in asthma. Am J Respir Crit Care Med. 2004;170:148‐153. 15070820 10.1164/rccm.200309-1304OC

[52] Ckless K , Lampert A , Reiss J , et al. Inhibition of arginase activity enhances inflammation in mice with allergic airway disease, in association with increases in protein S‐nitrosylation and tyrosine nitration. J Immunol. 2008;181:4255‐4264. 18768883 10.4049/jimmunol.181.6.4255 PMC2892856

[53] Xu W , Ghosh S , Comhair SAA , et al. Increased mitochondrial arginine metabolism supports bioenergetics in asthma. J Clin Invest. 2016;126:2465‐2481. 27214549 10.1172/JCI82925 PMC4922712

[54] Iglesias DE , Bombicino SS , Valdez LB , Boveris A . Nitric oxide interacts with mitochondrial complex III producing antimycin‐like effects. Free Radic Biol Med. 2015;89:602‐613. 26456055 10.1016/j.freeradbiomed.2015.08.024

[55] Ahmad T , Mabalirajan U , Ghosh B , Agrawal A . Altered asymmetric dimethyl arginine metabolism in allergically inflamed mouse lungs. Am J Respir Cell Mol Biol. 2010;42:3‐8. 19648472 10.1165/rcmb.2009-0137RC

[56] Holguin F , Comhair SAA , Hazen SL , et al. An association between L‐arginine/asymmetric dimethyl arginine balance, obesity, and the age of asthma onset phenotype. Am J Respir Crit Care Med. 2013;187:153‐159. 23204252 10.1164/rccm.201207-1270OC PMC3570651

[57] Liu G , Summer R . Cellular metabolism in lung health and disease. Annu Rev Physiol. 2019;81:403‐428. 30485759 10.1146/annurev-physiol-020518-114640 PMC6853603

[58] Palsson‐McDermott EM , O'Neill LAJ . The Warburg effect then and now: from cancer to inflammatory diseases. Bioessays. 2013;35:965‐973. 24115022 10.1002/bies.201300084

[59] Loftus RM , Finlay DK . Immunometabolism: cellular metabolism turns immune regulator. J Biol Chem. 2016;291:1‐10. 26534957 10.1074/jbc.R115.693903 PMC4697146

