# Supplementary material for: Comparative metabolomics analysis of bronchial epithelium during barrier establishment after allergen exposure
Source: Clin Transl Allergy. 2021 Sep 21;11(7):e12051. doi: 10.1002/clt2.12051 (PMC9082991; doi:10.1002/clt2.12051)
Supplement: Supplementary file 1 — Supporting Information 1 [file CLT2-11-e12051-s001.docx]

**Supporting Information**

**Comparative metabolomics analysis of bronchial epithelium during barrier establishment after allergen exposure**

**Running title:** Epithelial barrier metabolome in allergen exposure

López-Rodríguez Juan Carlos^1,a^, Rodríguez-Coira Juan^2,3,a^, Benedé Sara^1^, Barbas Coral^2^, Barber, Domingo^3^, Villalba Mayte^1^, Escribese María Marta^3^, Villaseñor Alma^2,3*^, Batanero Eva^1,*^

^1^Departamento de Bioquímica y Biología Molecular, Facultad de Ciencias Químicas. Universidad Complutense de Madrid, Madrid, Spain;

^2^Centro de Metabolómica y Bioanálisis (CEMBIO), Facultad de Farmacia, Universidad San Pablo-CEU, CEU Universities, Madrid, Spain;

^3^Instituto de Medicina Molecular Aplicada (IMMA), Departamento de Ciencias Médicas Básicas. Facultad de Medicina. Universidad San Pablo-CEU, CEU Universities, Madrid, Spain

^a^These authors have equally contributed to this work.

*Both authors act as equivalent co-senior authors and corresponding authors:

Eva Batanero, PhD ([ebataner@ucm.es](mailto:ebataner@ucm.es)). Departamento de Bioquímica y Biología Molecular, Facultad de Ciencias Químicas. Universidad Complutense. Madrid (Spain)

Alma Villaseñor, PhD (alma.villasenor@ceu.es).Centro de Metabolómica y Bioanálisis (CEMBIO), Facultad de Farmacia, Universidad San Pablo-CEU, CEU Universities, Madrid (Spain)

**MATERIALS AND METHODS**

**Immunofluorescence labelling and confocal laser scanning microscopy of tight junctions**

After allergen exposure, ALI-cultured Calu-3 cells were washed with PBS, fixed with 4% (w/v) paraformaldehyde (Sigma) in PBS for 15 min, and permeabilized with 0.05% (v/v) Triton-X100 (Sigma). Samples were blocked with 3% (w/v) bovine serum albumin (BSA, Sigma) in PBS containing 0.05% Tween-20 (Sigma), and incubated with rabbit polyclonal anti-ZO-1 (1/75, Invitrogen) for TJs subcellular localization, and a goat anti-rabbit IgG labelled with Alexa 647 (1/400, Invitrogen, CA, USA) as secondary antibody. After staining, membranes were removed from the transwell inserts using a sterile scalpel and mounted onto glass slides (Deltalab, Barcelona, Spain) with ProLongTM Gold antifade mountant containing DAPI (4’,6-Diamidino-2-Phenylindole Dihydrochloride, Thermo Fischer Scientific). Images were obtained with an OLYMPUS FV1200 confocal laser microscope at the Centro de Citometría y Microscopía de Fluorescencia (CAI-UCM). Experiments were performed in duplicate and representative images are shown.

**Metabolomics analysis**

The apical and basolateral media were analysed using a multiplatform platforms: liquid chromatography-quadrupole time-of-flight mass spectrometry (LC-QTOF-MS) and capillary electrophoresis-time-of-flight mass spectrometry (CE-TOF-MS).

**Sample preparation**

For LC-QTOF-MS, proteins were removed by adding 1 volume of the extracellular media to 3 volumes of cold methanol (-20 °C). Then, samples were vortex-mixed, incubated at -20°C for 5 min, and centrifuged at 16000 x*g* for 20 min at 4°C. Supernatant containing the metabolites were transferred to a LC vial (Agilent Technologies, USA) for analysis.

For CE-TOF-MS, an aliquot of extracellular media (200 μL of basolateral and 80 μL of apical media) were mixed with 5μL of acetonitrile containing formic acid (4.1 mM), and methionine sulfone as internal standard (final concentration 22.5 mM).^1,2^ In case of apical samples, 70 μL of water were added to each sample to reach minimum volume of ultrafiltration device (150 μL). Then, samples were vortex-mixed and ultrafiltered using a Centrifree® 30 kDa cut-off ultrafiltration device (Millipore Ireland Ltd., Cork, Ireland) at 2000 x*g* for 80 minutes at 4 °C to remove higher molecular mass proteins. The filtrates were transferred to CE vials (Agilent Technologies, USA) for analysis.

**Quality control preparation**

For each analytical platform, independent quality controls (QC) were prepared by pooling equal volumes of apical and basolateral media. The QC samples were then treated following the procedure described above. QC samples were analyzed throughout the worklist sequence to provide evidence about stability, performance and reproducibility of the analytical technique.

**Analytical platforms**

Samples were analyzed in separate runs in a randomized order for the corresponding analytical technique.

*a)* For LC-QTOF-MS analysis, an Agilent 1200 series HPLC system (Agilent Technologies, Waldbronn, Germany) coupled to an Agilent 6520 series Q-TOF-MS system was used as previously described.^3^

Briefly, LC was equipped with a degasser, two binary pumps, and a thermostated autosampler. For the separation, 10 μL of sample were injected into a Zorbax Eclipse XDB-C18 (4.6 mm × 50 mm, 1.8 µm, Agilent Technologies) column, with a temperature set at 40°C. The flow rate was 0.5 mL/min, using as mobile phases water containing 0.1% (v:v) formic acid (solvent A), and methanol (solvent B). The gradient elution was 5 min of isocratic 2% B(0–5 min), a linear gradient to 50% B in 9 min (6–15 min), 2 min hold at 50% B (15–17 min), and then a 1 min-linear gradient to 95% B (17–18 min), hold at 95% B for 2 min (18–20 min) before returning to start conditions. Finally, the column was re-equilibrated with 2%B for 5 min (20-25 min).

The QTOF-MS system (Agilent Technologies), was equipped with a dual electrospray ionization (ESI) source. Mass spectra were acquired in both positive (ESI+) and negative (ESI-) ionization modes with the range set at *m/z* 100 to 1100 and a scan rate of 1 scan/s. Internal reference masses in ESI+ mode were set at *m/z* 121.0509 (for purine) and *m/z* 922.0098 (for HP-921: hexakis-(1H,1H,3H-tetrafluoro-pentoxy)phosphazene), whereas in ESI- mode were set at *m/z* 112.9856 (for trifluoroacetic acid) and *m/z* 922.009798 (for HP-921). The conditions used were capillary voltages of 3500 V and 4000 V for positive and negative ionization modes, respectively, a nebulizer pressure of 35 psi, a drying gas flow of 11 L/min, a fragmentor voltage of 75V and a radiofrequency voltage in the octupole of 750 V.

*b)* For CE-TOF-MS, an Agilent 7100 series CE system coupled to an Agilent 6224 series TOF/MS system was used as previously described.^1,2^

CE-TOF-MS was conducted in ESI+ mode, and spectra was acquired with a full scan in the *m/z* range of 80-1000 at a rate of 1.4 scans/s. Separation was carried out using a 100 cm fused-silica capillary (50 μm internal diameter) (Agilent technologies). The capillary was pre-conditioned with 1 M NaOH for 30 min, followed by MilliQ® water and background electrolyte-BGE (0.8 M of FA in 10% methanol) for 30 min, at 20°C, using normal polarity. The conditions used were a drying gas flow of 10 L/min, a nebulizer pressure of 10 psi, a capillary voltage of 3.5 kV, a fragmentor voltage of 125 V, a gas temperature of 200ºC and skimmer of 65 V. Before each run, the capillary was washed with BGE for 5 min, using a 950 mbar pressure, and samples were injected at 50 mbar for 50 s (stacked by applying BGE at 100 mbar for 10 s), and a voltage of 30 kV with 25 mbar of internal pressure was applied. Sheath liquid composition was 1:1 (v/v) methanol:water containing 1.0 mM of formic acid, and purine (121.0509 *m/z*) and HP-921 (922.0098 *m/z*) as internal reference masses for the calibration of the instrument.

**Data treatment**

Raw data were extracted and analyzed with MassHunter Profinder software (B.08.00, Agilent Technologies), and consisted of two consecutive algorithms. First, the molecular feature extraction algorithm, which reduces the data size and complexity by removing associated non-specific information and extracting important features; and second, the find by ion algorithm, which improves the accuracy of the data, was used to perform a targeted feature extraction. Finally, peak area, mass and retention time for each feature in all samples were obtained in a matrix data form. Data quality was assured excluding background noises and unrelated ions by keeping molecular features present in the 50% of QC injections with a coefficient of variation (CV) below 30%, and present in 75% of the samples of the study. Missing values were estimated using k-nearest neighbors algorithm.

**Metabolite annotation**

A tentative identification of all significant features was performed through the CEU Mass Mediator 3.0 in-house software.^4^

For LC-QTOF-MS, identification was confirmed by LC-MS/MS, using a 20V fragmentation energy. If available, the MS/MS spectra were compared against Human Metabolome Database (HMDB), Kyoto Encyclopedia of Genes and Genomes (KEGG), LIPID MAPS 1.0.54 (Agilent Technologies) and METLIN databases. For CE-TOF-MS, if available, the identity of the compound was assessed by spiking the commercial standard to the sample, and checking the increment of the signal compared against sample and standard alone.^1^

**Statistical analysis**

Data were analysed using the multivariate statistical analysis SIMCA P+ 14.0.1 (Sartorius, Göttingen, Germany) software. Data quality, outliers’ detection and identification of sample patterns were performed using a non-supervised model by principal components analysis (PCA).^5^ Additionally, the partial least squares discriminant analysis (PLS-DA) was used to confirm the separation between groups. For all models unit variance-scaling was applied.^5^

Univariate statistics analysis were performed using the Mass Profiler Professional MPP v14.9.1 (Agilent technologies) software. Thus, non-parametric Mann-Whitney U was used to determine statistical significance between pair groups (p < 0.05). Data were corrected by false discovery rate (FDR) < 0.1.

Heatmaps were obtained using Metaboanalyst 4.0 software ^6^, calculating the Euclidean distance between the samples, and Ward’s algorithm to calculate similarity between clusters.

The percentage of change was calculated as follows: % Change = [(average value in treated group − average value in control group)/(average value in control group)] x 100.

**Pathway analysis**

Pathways of the significant identified compounds were obtained using IMPaLA (v 12.0) online tool (<http://impala.molgen.mpg.de/>), which uses the pathways from KEEG and Reactome databases.

**REFERENCES**

1. Mamani-Huanca M, Gradillas A, Gil de la Fuente A, López-Gonzálvez Á, Barbas C. Unveiling the Fragmentation Mechanisms of Modified Amino Acids as the Key for Their Targeted Identification. *Anal Chem*. 2020;92:4848-4857.

2. Canuto GAB, Castilho-Martins EA, Tavares M, López-Gonzálvez A, Rivas L, Barbas C. CE-ESI-MS metabolic fingerprinting of Leishmania resistance to antimony treatment. *Electrophoresis*. 2012;33:1901-1910.

3. Ciborowski M, Javier Rupérez F, Martínez-Alcázar MP, et al. Metabolomic approach with LC-MS reveals significant effect of pressure on diver’s plasma. *J Proteome Res*. 2010;9:4131-4137.

4. Gil-de-la-Fuente A, Godzien J, Saugar S, et al. CEU Mass Mediator 3.0: A Metabolite Annotation Tool. *J Proteome Res*. 2019;18:797-802.

5. Smith PF. On the Application of Multivariate Statistical and Data Mining Analyses to Data in Neuroscience. *J Undergrad Neurosci Educ*. 2018;16:R20-R32.

6. Chong J, Wishart DS, Xia J. Using MetaboAnalyst 4.0 for Comprehensive and Integrative Metabolomics Data Analysis. *Curr Protoc Bioinformatics*. 2019;68:e86.

| **Table S1.** Significantly different metabolites in the apical media of ALI-cultured Calu-3 cells exposed to Der p 1 or Ole e 1, on day 2 of culture. | | | | | | | | | | | | |
| --- | --- | --- | --- | --- | --- | --- | --- | --- | --- | --- | --- | --- |
| **Nº** | **Metabolite^1^** | **Accession number^2^** | **Mass (Da)^3^** | **Adduct** | **RT (min)^4^** | **Chemical formula** | **Error (ppm)** | **Platform^5^** | **CV in QC (%)^6^** | **Biochemical subclass** | **Change (%)^7^** | |
|  |  |  |  |  |  |  |  |  |  |  | **Der p 1** | **Ole e 1** |
| 1 | Acetylgalactosamine | HMDB0000212 | 221.0900 | M+H | 26.29 | C_8_H_15_NO_6_ | 0 | CE-MS | 3.22 | Carbohydrate and carbohydrate conjugate | 18.1* | 15.9 |
| 2 | L-Alanine† | HMDB0000161 | 89.0482 | M+H | 13.07 | C_3_H_7_NO_2_ | 6 | CE-MS | 2.91 | Amino acid, peptides and analogues | 93.4* | 37.4 |
| 3 | Amino-dodecanoic acid | LMFA01100005 | 215.1854 | M+Na | 20.48 | C_12_H_25_NO_2_ | 15 | LC-MS+ | 25.33 | Amino fatty acid | 104.0* | 19.7 |
| 4 | L-Arginine† | HMDB00517 | 174.1120 | M+H | 10.93 | C_6_H_14_N_4_O_2_ | 4 | CE-MS | 2.23 | Amino acid, peptides and analogues | 86.6* | 25.0 |
| 5 | L-Asparagine† | HMDB00168 | 132.0525 | M+H | 14.64 | C_4_H_8_N_2_O_3_ | 7 | CE-MS | 5.78 | Amino acid, peptides and analogues | 89.9* | 33.0 |
| 6 | Aspartylglycylaspartate | 16992 | 305.0864 | M-H | 19.51 | C_10_H_15_N_3_O_8_ | 2 | LC-MS- | 7.97 | Amino acid, peptides and analogues | a* | a* |
| 7 | Aspartylvaline  Glutamylamino butanoate  Hydroxyprolyl-threonine | HMDB28766  HMDB12161  HMDB28873 | 232.1077 | M+H | 15.67 | C_9_H_16_N_2_O_5_ | 8 | LC-MS+ | 27.77 | Amino acid, peptides and analogues | 54.8* | 28.8* |
| 8 | Betaine† | HMDB0000043 | 117.0788 | M+H | 15.64 | C_5_H_11_NO_2_ | 1 | CE-MS | 9.08 | Amino acid, peptides and analogues | 49.1* | 21.5 |
| 9 | N-(1-Deoxy-1-fructosyl)leucine  N-(1-Deoxy-1-fructosyl)isoleucine | HMDB37840  HMDB0039780 | 293.1477 | M+H | 18.23 | C_12_H_23_NO_7_ | 1 | CE-MS | 3.46 | Amino acid, peptides and analogues | 84.3* | 22.1 |
| 10 | Desaminotyrosine | HMDB0002199 | 166.0668 | M-H-H_2_O | 9.64 | C_9_H_10_O_3_ | 2 | LC-MS- | 13.73 | Phenylpropanoic acid | a* | a* |
| 11 | D-Fructose (or any hexose isomer) | HMDB0000660 | 180.0623 | M-H | 1.96 | C_6_H_12_O_6_ | 6 | LC-MS- | 19.08 | Carbohydrate and carbohydrate conjugate | 96.9* | 32.1 |
| 12 | Folinic acid | HMDB0001562 | 471.1433 | M+Na | 20.04 | C_20_H_23_N_7_O_7_ | 2 | LC-MS+ | 7.69 | Pterins and derivatives | 75.0* | -57.7* |
| 13 | Fructosyl-lysine | HMDB0034879 | 308.1598 | M+H | 12.96 | C_12_H_24_N_2_O_7_ | 5 | CE-MS | 10.20 | Carbohydrate and carbohydrate conjugate | 179.5* | 49.4 |
|  | | | | | | | | | | | | |
| **Table S1 (continuation).** Significantly different metabolites in the apical media of ALI-cultured Calu-3 cells exposed to Der p 1 or Ole e 1, on day 2 of culture. | | | | | | | | | | | | |
| **Nº** | **Metabolite^1^** | **Accession number^2^** | **Mass (Da)^3^** | **Adduct** | **RT (min)^4^** | **Chemical formula** | **Error (ppm)** | **Platform^5^** | **CV in QC (%)^6^** | **Biochemical subclass** | **Change (%)^7^** | |
|  |  |  |  |  |  |  |  |  |  |  | **Der p 1** | **Ole e 1** |
| 14 | L-Glutamic acid† | HMDB0000148 | 147.0542 | M+H | 15.27 | C_5_H_9_NO_4_ | 6 | CE-MS | 8.51 | Amino acid, peptides and analogues | 257.8* | -51.5 |
| 15 | Glycerophosphocholine | HMDB0000086 | 257.1031 | M+H | 24.96 | C_8_H_20_NO_6_P | 1 | CE-MS | 4.00 | Glycerophosphocholines | -26.7* | -4.2 |
| 16 | L-Histidine† | HMDB0000177 | 155.0696 | M+H | 11.15 | C_6_H_9_N_3_O_2_ | 1 | CE-MS | 3.10 | Amino acid, peptides and analogues | 133.4* | 21.6 |
| 17 | Hydroxy-L-tryptophan | HMDB0000472 | 220.0858 | M+H | 17.4 | C_11_H_12_N_2_O_3_ | 5 | CE-MS | 3.55 | Tryptamines and derivatives | 56.2* | 12.4 |
| 18 | L-Isoleucine†  L-Leucine† | HMDB0000172  HMDB0000687 | 131.0947 | M+H | 14.46 | C_6_H_13_NO_2_ | 1 | CE-MS | 14.04 | Amino acid, peptides and analogues | 201.9* | 24.4 |
| 19 | L-Kynurenine† | HMDB0000684 | 208.0848 | M+H | 14.32 | C_10_H_12_N_2_O_3_ | 0 | CE-MS | 2.98 | Carbonyl compound | 185.2* | 21.0 |
| 20 | LacCer(d18:0/26:0) | LMSP0501AB19 | 1003.7851 | M-H | 1.62 | C_56_H_109_NO_13_ | 5 | LC-MS- | 3.47 | Sphingolipids | -28.5 | -28.8* |
| 21 | L-Lysine† | HMDB0000182 | 146.1060 | M+H | 10.61 | C_6_H_14_N_2_O_2_ | 3 | CE-MS | 2.69 | Amino acid, peptides and analogues | 70.9* | 26.5 |
| 22 | Methionine sulfoxide† | HMDB0002005 | 165.0460 | M+H | 17.71 | C_5_H_11_NO_3_S | 2 | CE-MS | 2.62 | Amino acid, peptides and analogues | 92.9* | 27.7 |
| 23 | L-Methionine† | HMDB0000696 | 149.0521 | M+H | 14.88 | C_5_H_11_NO_2_S | 7 | CE-MS | 1.75 | Amino acid, peptides and analogues | 208.4* | 18.4 |
| 24 | Methylated-tyrosyltryptophan-OH | 65040 | 503.1712 | M-H | 20.05 | C_27_H_25_N_3_O_7_ | 4 | LC-MS- | 15.78 | Amino acid, peptides and analogues | 80.9* | 56.1 |
| 25 | Methylnicotinamide | HMDB0059711 | 136.0635 | M+H | 11.07 | C_7_H_10_N_4_O | 2 | CE-MS | 3.11 | Pyridinecarboxylic acids and derivatives | -19.1* | 7.2 |
| 26 | Oxoisopentadecanoyl-CoA | LMFA07050246 | 1005.2930 | M-H | 1.7 | C_36_H_62_N_7_O_18_P_3_S | 15 | LC-MS- | 10.07 | Fatty acyl CoAs | -45.3* | -29.1 |
| 27 | Pantothenic acid† | HMDB00210 | 219.1111 | M+H | 25.29 | C_9_H_17_NO_5_ | 2 | CE-MS | 3.28 | Alcohols and polyols | 47.6* | 13.9 |
| 28 | PE(38:9) | LMGP02010762 | 757.4671 | M+Na | 0.95 | C_43_H_68_NO_8_P | 2 | LC-MS+ | 7.91 | Diacylglycerophospho-  ethanolamines | -38.7* | -22.0 |
|  |  |  |  |  |  |  |  |  |  |  |  |  |
| **Table S1 (continuation).** Significantly different metabolites in the apical media of ALI-cultured Calu-3 cells exposed to Der p 1 or Ole e 1, on day 2 of culture. | | | | | | | | | | | | |
| **Nº** | **Metabolite^1^** | **Accession number^2^** | **Mass (Da)^3^** | **Adduct** | **RT (min)^4^** | **Chemical formula** | **Error (ppm)** | **Platform^5^** | **CV in QC (%)^6^** | **Biochemical subclass** | **Change (%)^7^** | |
|  |  |  |  |  |  |  |  |  |  |  | **Der p 1** | **Ole e 1** |
| 29 | L-Phenylalanine† | HMDB0000159 | 165.0801 | M+H | 15.29 | C_9_H_11_NO_2_ | 6 | CE-MS | 5.18 | Amino acids, peptides, and analogues | 190.2* | 19.6 |
| 30 | L-Serine† | HMDB00187 | 105.0426 | M+H | 15.17 | C_3_H_7_NO_3_ | 1 | CE-MS | 3.10 | Amino acid, peptides and analogues | 97.1* | 28.5 |
| 31 | Stearidonoyl-CoA | HMDB06519 | 1025.3061 | M-H-H_2_O | 1.71 | C_39_H_62_N_7_O_17_P_3_S | 11 | LC-MS- | 11.49 | Fatty acyl thioesters | -34.3* | -17.9 |
| 32 | L-Tryptophan† | HMDB00929 | 204.0904 | M+H | 15.23 | C_11_H_12_N_2_O_2_ | 3 | CE-MS | 2.27 | Amino acid, peptides and analogues | 201.2* | 18.5 |
| 33 | Tyramine | HMDB00306 | 138.0955 | M+H-H_2_O | 6.49 | C_8_H_11_NO | 3 | LC-MS+ | 11.61 | Phenethylamines | 174.1* | 21.0 |
| 34 | L-Tyrosine† | HMDB00158 | 181.0741 | M+H | 15.61 | C_9_H_11_NO_3_ | 1 | CE-MS | 3.04 | Amino acid, peptides and analogues | 224.8* | 64.5 |
| 35 | L-Valine† | HMDB00883 | 117.0796 | M+H | 14.14 | C_5_H_11_NO_2_ | 6 | CE-MS | 2.76 | Amino acid, peptides and analogues | 146.7* | 21.3 |
| ^1^Metabolite: †, annotation was done by spiking of the commercial standard; ‡, annotation was carried out by comparison of MS/MS experiments. PE, phosphatidylethanolamine. The annotation for PE is the total number of carbons from the fatty acids chains: and the number of double bonds.  ^2^C, KEGG; HMDB, Human Metabolome Database; LM, LIPID MAPS; Number, METLIN.  ^3^Experimental mass.  ^4^RT, retention time.  ^5^Percentage (%) of coefficient of variation (CV) in quality control (QC).  ^6^CE-MS, capillary electrophoresis-time-of-flight mass spectrometry; LC-MS, liquid chromatography-quadrupole time-of-flight mass spectrometry.  ^7^Percentage (%) of change: (*), p<0.05; Positive value, increase; Negative value, decrease. a, only in allergen-exposed cultures. | | | | | | | | | | | | |

| **Table S2.** Significantly different metabolites in the apical media of ALI-cultured Calu-3 cells exposed to Der p 1 or Ole e 1, on day 7 of culture. | | | | | | | | | | | | |
| --- | --- | --- | --- | --- | --- | --- | --- | --- | --- | --- | --- | --- |
| **Nº** | **Metabolite^1^** | **Accession number^2^** | **Mass (Da)^3^** | **Adduct** | **RT (min)^4^** | **Chemical formula** | **Error (ppm)** | **Platform^5^** | **CV in QC (%)^6^** | **Biochemical class** | **Change (%)^7^** | |
|  |  |  |  |  |  |  |  |  |  |  | **Der p 1** | **Ole e 1** |
| 1 | Acetamidobutanal  Pipecolic acid | HMDB59649  HMDB0000070 | 129.0789 | M+H | 0.86 | C_6_H_11_NO_2_ | 0 | LC-MS+ | 5.90 | Amino acids, peptides, and analogues | 266.0* | 41.3 |
| 2 | L-Alanine† | HMDB0000161 | 89.0482 | M+H | 13.07 | C_3_H_7_NO_2_ | 6 | CE-MS | 2.91 | Amino acids, peptides, and analogues | 949.5* | 31.9 |
| 3 | L-Arginine† | HMDB00517 | 174.1120 | M+H | 10.93 | C_6_H_14_N_4_O_2_ | 2 | CE-MS | 2.23 | Amino acids, peptides, and analogues | 774.5* | 37.3 |
| 4 | L-Asparagine† | HMDB00168 | 132.0525 | M+H | 14.64 | C_4_H_8_N_2_O_3_ | 7 | CE-MS | 5.78 | Amino acids, peptides, and analogues | 875.6* | 42.6 |
| 5 | Asymmetric dimethylarginine | HMDB01539 | 202.1422 | M+H | 11.61 | C_8_H_18_N_4_O_2_ | 4 | CE-MS | 17.66 | Amino acids, peptides, and analogues | 530.6* | 51.3 |
| 6 | L-Cysteinylglycine disulfide | HMDB00709 | 297.0450 | M+H | 13.71 | C_8_H_15_N_3_O_5_S_2_ | 1 | CE-MS | 2.66 | Amino acids, peptides, and analogues | 494.9* | 305.9* |
| 7 | L-Cystine† | HMDB00192 | 240.0249 | M+H | 15.35 | C_6_H_12_N_2_O_4_S_2_ | 4 | CE-MS | 3.20 | Amino acids, peptides, and analogues | 268.9* | 20.2* |
| 8 | Desaminotyrosine | HMDB0002199 | 166.0668 | M-H-H20 | 9.64 | C_9_H_10_O_3_ | 2 | LC-MS- | 13.73 | Amino acids, peptides, and analogues | a* | 0 |
| 9 | Diaminohexanoic acid | HMDB0142894 | 146.1061 | M+H | 15.85 | C_6_H_14_N_2_O_2_ | 4 | LC-MS+ | 7.08 | Amino acids, peptides, and analogues | 147.6* | 36.6 |
| 10 | L-Glutamine† | HMDB00641 | 146.0697 | M+H | 14.98 | C_5_H_10_N_2_O_3_ | 4 | CE-MS | 1.33 | Amino acids, peptides, and analogues | 1284.0* | 35.3 |
| 11 | Glutaminylglutaminylcystine | 17762 | 377.1237 | M+K | 16.57 | [C_13_H_23_N_5_O_6_S](https://pubchem.ncbi.nlm.nih.gov/#query=C13H23N5O6S) | 14 | LC-MS+ | 5.49 | Amino acids, peptides, and analogues | 355.1* | 34 |
| 12 | Glycine† | HMDB00123 | 75.0328 | M+H | 12.99 | C_2_H_5_NO_2_ | 0 | CE-MS | 1.49 | Amino acids, peptides, and analogues | 534.3* | 38.2 |
| 13 | L-Histidine† | HMDB0000177 | 155.0696 | M+H | 11.15 | C_6_H_9_N_3_O_2_ | 1 | CE-MS | 3.10 | Amino acids, peptides, and analogues | 720.1* | 32.4 |
|  |  |  |  |  |  |  |  |  |  |  |  |  |
| **Table S2 (continuation).** Significantly different metabolites in the apical media of ALI-cultured Calu-3 cells exposed to Der p 1 or Ole e 1, on day 7 of culture. | | | | | | | | | | | | |
| **Nº** | **Metabolite^1^** | **Accession number^2^** | **Mass (Da)^3^** | **Adduct** | **RT (min)^4^** | **Chemical formula** | **Error (ppm)** | **Platform^5^** | **CV in QC (%)^6^** | **Biochemical class** | **Change (%)^7^** | |
|  |  |  |  |  |  |  |  |  |  |  | **Der p 1** | **Ole e 1** |
| 14 | Homovanillic acid  Phenylpyruvic acid | HMDB00118  HMDB0000205 | 182.0570  164.0465 | M+H-H_2_O  M+H | 2.81 | C_9_H_10_O_4_  C_9_H_8_O_3_ | 5 | LC-MS+ | 5.58 | Methoxyphenols | 484.7* | -2.6 |
| 15 | Inositol -pentakisphosphate | C04579 | 579.9048 | M-H-H_2_O | 1.76 | C_6_H_17_O_21_P_5_ | 10 | LC-MS- | 10.44 | Inositol phosphate | -82.5* | -59.4 |
| 16 | L-Isoleucine† | HMDB0000172 | 131.0947 | M+H | 14.46 | C_6_H_13_NO_2_ | 1 | CE-MS | 3.91 | Amino acids, peptides, and analogues | 1423.5* | 46.5 |
| 17 | Lactoylleucine | HMDB0062176 | 203.1151 | M-H | 18.57 | C_9_H_17_NO_4_ | 3 | LC-MS- | 16.62 | Amino acids, peptides, and analogues | 118.4* | -24.5 |
| 18 | L-Lysine† | HMDB0000182 | 146.1057 | M+H | 10.61 | C_6_H_14_N_2_O_2_ | 3 | CE-MS | 2.69 | Amino acids, peptides, and analogues | 823.8* | 43.9 |
| 19 | Methionine sulfoxide† | HMDB0002005 | 165.0463 | M+H | 17.71 | C_5_H_11_NO_3_S | 2 | CE-MS | 2.62 | Amino acids, peptides, and analogues | a* | a* |
| 20 | L-Methionine† | HMDB0000696 | 149.0521 | M+H | 14.88 | C_5_H_11_NO_2_S | 7 | CE-MS | 1.75 | Amino acids, peptides, and analogues | 888.8* | 33.9 |
| 21 | Methionylasparaginylasparagine | 15896 | 377.1237 | M+K | 16.57 | [C_13_H_23_N_5_O_6_S](https://pubchem.ncbi.nlm.nih.gov/#query=C13H23N5O6S) | 14 | LC-MS+ | 5.49 | Amino acids, peptides, and analogues | 355.1* | 34 |
| 22 | Methionylmethionyltryptophan | 17125 | 466.1644 | M+H | 20.61 | C_21_H_30_N_4_O_4_S_2_ | 14 | LC-MS+ | 6.88 | Amino acids, peptides, and analogues | 232.3* | 139.4* |
| 23 | Methylated-tyrosyltrytophan-OH | 65040 | 503.1712 | M-H | 20.05 | C_27_H_25_N_3_O_7_ | 4 | LC-MS- | 15.78 | Amino acids, peptides, and analogues | b* | b* |
| 24 | Heptanoylglycine | HMDB0013010 | 187.1199 | M-H | 17.47 | C_9_H_17_NO_3_ | 5 | LC-MS- | 14.58 | Amino acids, peptides, and analogues | -3.5 | 25.1* |
| 25 | Niacinamide | HMDB0001406 | 122.0483 | M+H | 11.27 | C_6_H_6_N_2_O | 2 | CE-MS | 4.77 | Pyridinecarboxylic acids and derivatives | 58.1 | 81.3* |
| 26 | Oxaloglutarate | C05533 | 204.0270 | M-H | 1.95 | C_7_H_8_O_7_ | 0 | LC-MS- | 5.82 | Tricarboxylic acid | -56.1 | -59.7* |
| 27 | L-Phenylalanine† | HMDB0000159 | 165.0801 | M+H | 15.29 | C_9_H_11_NO_2_ | 7 | CE-MS | 2.70 | Amino acids, peptides, and analogues | 1226.3* | 36.8 |
| **Table S2 (continuation).** Significantly different metabolites in the apical media of ALI-cultured Calu-3 cells exposed to Der p 1 or Ole e 1, on day 7 of culture. | | | | | | | | | | | | |
| **Nº** | **Metabolite^1^** | **Accession number^2^** | **Mass (Da)^3^** | **Adduct** | **RT (min)^4^** | **Chemical formula** | **Error (ppm)** | **Platform^5^** | **CV in QC (%)^6^** | **Biochemical class** | **Change (%)^7^** | |
|  |  |  |  |  |  |  |  |  |  |  | **Der p 1** | **Der p 1** |
| 28 | PI(37:7) | LMGP06010791 | 866.4856 | M-H | 1.73 | [C_46_H_75_O_13_P](https://www.lipidmaps.org/tools/ms/iso2d_Ag.php?formula=C46H75O13P) | 10 | LC-MS- | 4.04 | Diacylglycerophosphoinositols | -20.8 | -41.8* |
| 29 | L-Proline† | HMDB0000162 | 115.0632 | M+H | 15.05 | C_5_H_9_NO_2_ | 1 | CE-MS | 1.89 | Amino acids, peptides, and analogues | 231.9* | 46 |
| 31 | Prolylphenylalanine | HMDB0011179 | 262.1315 | M+H | 12.29 | C_14_H_18_N_2_O_3_ | 1 | CE-MS | 22.99 | Amino acids, peptides, and analogues | a* | a* |
| 32 | PS(41:4) | LMGP03010478 | 853.6003 | M-H | 1.68 | [C_47_H_84_NO_10_P](https://www.lipidmaps.org/tools/ms/iso2d_Ag.php?formula=C47H84NO10P) | 20 | LC-MS- | 6.87 | Diacylglycerophosphoserines | -36 | -81.8* |
| 33 | L-Serine† | HMDB0000187 | 105.0426 | M+H | 15.17 | C_3_H_7_NO_3_ | 1 | CE-MS | 3.10 | Amino acids, peptides, and analogues | 797.0* | 11 |
| 34 | Succinylacetoacetate | HMDB0240258 | 202.0445 | M-H | 2.37 | C_8_H_10_O_6_ | 16 | LC-MS- | 18.71 | Medium-chain keto acids and derivatives | 95.3* | -2.7 |
| 35 | Stearidonoyl-CoA | HMDB06519 | 202.0445 | M-H | 2.37 | C_8_H_10_O_6_  C_39_H_62_N_7_O_17_P_3_S | 16 | LC-MS- | 18.71 | Medium-chain keto acids and derivatives  Fatty acyl thioesters | 95.3* | -2.7 |
| 36 | L-Threonine† | HMDB0000167 | 119.0572 | M+H | 14.69 | C_4_H_9_NO_3_ | 9 | CE-MS | 4.92 | Amino acids, peptides, and analogues | 776.6* | 35.8 |
| 37 | L-Tryptophan | HMDB00929 | 204.0904 | M+H | 15.23 | C_11_H_12_N_2_O_2_ | 3 | CE-MS | 2.27 | Amino acids, peptides, and analogues | 852.7* | 33 |
| 38 | Tyramine | HMDB00306 | 138.0955 | M+H-H_2_O | 6.49 | C_8_H_11_NO | 3 | LC-MS+ | 11.61 | Phenethylamines | 507.6* | 9.9 |
| 39 | L-Tyrosine† | HMDB00158 | 181.0741 | M+H | 15.61 | C_9_H_11_NO_3_ | 1 | CE-MS | 3.00 | Amino acids, peptides, and analogues | 975.8* | 23.5 |
| 40 | L-Valine† | HMDB00883 | 117.0796 | M+H | 14.14 | C_5_H_11_NO_2_ | 6 | CE-MS | 2.76 | Amino acids, peptides, and analogues | 1561.8* | 30.3 |
| ^1^Metabolite: †, annotation by spiking of the commercial standard; ‡, annotation by comparison of MS/MS experiments. PI, phosphatidylinositol; PS, phosphatidylserine. The annotation for PI and PS is the total number of carbons from the fatty acids chains: and the number of double bonds.  ^2^C, KEGG; HMDB, Human Metabolome Database; LM, LIPID MAPS; Number, METLIN.  ^3^Experimental mass.  ^4^RT, retention time.  ^5^Percentage (%) of coefficient of variation (CV) in quality control (QC).  ^6^CE-MS, capillary electrophoresis-time-of-flight mass spectrometry; LC-MS, liquid chromatography-quadrupole time-of-flight mass spectrometry.  ^7^Percentage (%) of change: (*), p<0.05; Positive value, increase; Negative value, decrease. a, only in allergen-exposed cultures; b, only in control cultures. | | | | | | | | | | | | |

| **Table S3.** Significantly different metabolites in the basolateral media of ALI-cultured Calu-3 cells exposed to Der p 1 or Ole e 1, on day 2 of culture. | | | | | | | | | | | | |
| --- | --- | --- | --- | --- | --- | --- | --- | --- | --- | --- | --- | --- |
| **Nº** | **Metabolite^1^** | **Accession number^2^** | **Mass (Da)^3^** | **Adduct** | **RT (min)^4^** | **Chemical formula** | **Error (ppm)** | **Platform^5^** | **CV in QC (%)^6^** | **Biochemical class** | **Change (%)^7^** | |
|  |  |  |  |  |  |  |  |  |  |  | **Der p 1** | **Ole e 1** |
| 1 | Asparaginylalanine  Glutamylglycine | HMDB28724  HMDB28819 | 203.0904 | M+H | 0.83 | C_7_H_13_N_3_O_4_  C_7_H_12_N_2_O_5_ | 1 | LC-MS+ | 3.26 | Amino acids, peptides, and analogues | -5.3* | -4.2 |
| 2 | Glutamyltyrosyllysine  Histidylprolyltryptophan | 18989  23595 | 438.2042 | M+H-H_2_O | 0.82 | C_20_H_30_N_4_O_7_ | 11 | LC-MS+ | 4.66 | Amino acids, peptides, and analogues | -12.3* | -10.8* |
| 3 | Indolepyruvate | HMDB0060484 | 203.0485 | M+Na | 1.19 | C_11_H_9_NO_3_ | 13 | LC-MS+ | 5.45 | Indolyl carboxylic acids and derivatives | 0.8 | -12.5* |
| 4 | Leucylalanine | HMDB28922 | 202.1311 | M+H | 14.15 | C_9_H_18_N_2_O_3_ | 3 | LC-MS+ | 25.75 | Amino acids, peptides, and analogues | -22.2 | -46.2* |
| 5 | L-Lysine† | HMDB0000182 | 146.1056 | M+H | 10.67 | C_6_H_14_N_2_O_2_ | 1 | CE-MS | 8.74 | Amino acids, peptides, and analogues | -9.4* | 2.2 |
| 6 | Methylnicotinamide | HMDB00699 | 136.0635 | M+H | 11.07 | C_7_H_9_N_2_O | 2 | CE-MS | 3.11 | Pyridinecarboxylic acids and derivatives | 14.2* | 9.4 |
| 7 | Mevalonic acid | HMDB00227 | 148.0736 | M-H-H_2_O | 12.95 | C_6_H_12_O_4_ | 3 | LC-MS- | 3.64 | Fatty acids and conjugates | 266.0* | 16.5 |
| 8 | L-Phenylalanine† | HMDB0000159 | 165.0801 | M+H | 15.29 | C_9_H_11_NO_2_ | 6 | CE-MS | 5.18 | Amino acids, peptides, and analogues | -8.4* | 0.8 |
| 9 | Prolylglutamyltryptophan  Tyrosylthreonylphenylalanine | 21775  18327 | 429.1973 | M+Na | 20.19 | C_22_H_27_N_3_O_6_ | 17 | LC-MS+ | 12.16 | Amino acids, peptides, and analogues | 7.2 | 14.0* |
| ^1^Metabolite: †, annotation was done by spiking of the commercial standard; ‡, annotation was carried out by comparison of MS/MS experiments.  ^2^HMDB, Human Metabolome Database; LM, LIPID MAPS; Number, METLIN.  ^3^Experimental mass.  ^4^RT, retention time.  ^5^Percentage (%) of coefficient of variation (CV) in quality control (QC).  ^6^CE-MS, capillary electrophoresis-time-of-flight mass spectrometry; LC-MS, liquid chromatography-quadrupole time-of-flight mass spectrometry.  ^7^Percentage (%) of change: (*), p<0.05; Positive value, increase; Negative value, decrease. | | | | | | | | | | | | |

| **Table S4.** Significantly different metabolites in the basolateral media of ALI-cultured Calu-3 cells exposed to Der p 1 or Ole e 1, on day 7 of culture. | | | | | | | | | | | | |
| --- | --- | --- | --- | --- | --- | --- | --- | --- | --- | --- | --- | --- |
| **Nº** | **Metabolite^1^** | **Accession number^2^** | **Mass (Da)^3^** | **Adduct** | **RT (min)^4^** | **Chemical formula** | **Error (ppm)** | **Platform^5^** | **CV in QC (%)^6^** | **Biochemical class** | **Change (%)^7^** | |
|  |  |  |  |  |  |  |  |  |  |  | **Der p 1** | **Ole e 1** |
| 1 | Arginylprolylalanine (Arg Pro Ala) | 19199 | 324.1907 | M+H-H_2_O | 20.87 | C_14_H_26_N_6_O_4_ | 1 | LC-MS+ | 25.21 | Amino acids, peptides, and analogues | 26.7* | 27.3* |
| 2 | Asparaginylalanine (Asn Ala)  Glutamylglycine (Glu Gly) | HMDB28724  HMDB28819 | 203.0904 | M+H | 0.83 | C_7_H_12_N_2_O_5_ | 1 | LC-MS+ | 3.26 | Amino acids, peptides, and analogues | 13.3* | 5.5 |
| 3 | Dihydroxyoctadenoic acid  Dihydroxystearic acid | LMFA02000175  LMFA01050527 | 316.2614 | M+H | 20.75 | C_18_H_36_O_4_ | 0 | LC-MS+ | 17.68 | Other octadecanoids | 58.3* | 25 |
| 4 | L-Histidine† | HMDB0000177 | 155.0698 | M+H | 11.27 | C_6_H_9_N_3_O_2_ | 2 | CE-MS | 7.61 | Amino acids, peptides, and analogues | 10.0* | 12.0* |
| 5 | L-Lysine† | HMDB0000182 | 146.1056 | M+H | 10.67 | C_6_H_14_N_2_O_2_ | 1 | CE-MS | 8.74 | Amino acids, peptides, and analogues | 2.1 | 2.9* |
| 6 | Methionylisoleucine (Met Ile)  Methionylleucine (Met Leu) | HMDB28976  HMDB28977 | 262.1394 | M+H | 0.82 | C_11_H_22_N_2_O_3_S | 16 | LC-MS+ | 3.03 | Amino acids, peptides, and analogues | 8.0* | 3.4 |
| 7 | Mevalonic acid | HMDB00227 | 148.0730 | M-H- H_2_O | 12.95 | C_6_H_12_O_4_ | 3 | LC-MS- | 3.64 | Fatty acids and conjugates | -13.5* | -18.3* |
| 8 | L-Phenylalanine† | HMDB0000159 | 165.0777 | M+H | 15.45 | C_9_H_11_NO_2_ | 6 | CE-MS | 5.18 | Amino acids, peptides, and analogues | 1.7 | 5.1* |
| 9 | L-Tyrosine† | HMDB00158 | 181.0739 | M+H | 15.61 | C_9_H_11_NO_3_ | 0 | CE-MS | 4.72 | Amino acids, peptides, and analogues | 1.1 | 4.4* |
| 10 | Tyrosylasparagine-OH (Tyr-Asn-OH) | 65304 | 417.1179 | M+FA-H | 14.81 | C_19_H_19_N_3_O_8_ | 7 | LC-MS- | 2.74 | Amino acids, peptides, and analogues | 8.5* | 0.8 |
| ^1^Metabolite: †, annotation was done by spiking of the commercial standard; ‡, annotation was carried out by comparison of MS/MS experiments.  ^2^HMDB, Human Metabolome Database; LM, LIPID MAPS; Number, METLIN.  ^3^Experimental mass.  ^4^RT, retention time.  ^5^Percentage (%) of coefficient of variation (CV) in quality control (QC).  ^6^CE-MS, capillary electrophoresis-time-of-flight mass spectrometry; LC-MS, liquid chromatography-quadrupole time-of-flight mass spectrometry.  ^7^Percentage (%) of change: (*), p<0.05; Positive value, increase; Negative value, decrease. | | | | | | | | | | | | |

| **Table S5.** Significantly altered pathway in ALI-cultured Calu-3 cells exposed to Der p 1 or Ole e 1 along barrier establishment. | | | | | | | | | |
| --- | --- | --- | --- | --- | --- | --- | --- | --- | --- |
| **Pathway name** | **Source** | **Day 2** | | | | **Day 7** | | | |
|  |  | **Apical** | | **Basolateral** | | **Apical** | | **Basolateral** | |
|  |  | %^a^ | *p* value | %^a^ | *p* value | %^a^ | *p* value | %^a^ | *p* value |
| Alanine aspartate and glutamate metabolism | KEGG | 82.14 | 1.40 x 10^-3^ |  |  | 82.14 | 1.02 x 10^-3^ |  |  |
| Amine oxidase reactions | Reactome | 94.44 | 0.012 |  |  |  |  |  |  |
| Amine compound SLC transporters | Reactome |  |  | 88.57 | 0.0010 | 88.57 | 9.28 x 10^-23^ | 88.57 | 9.85 x 10^-6^ |
| Amine-derived hormones | Reactome | 97.37 | 4.26 x 10^-4^ |  |  | 97.37 | 4.12 x 10^-3^ |  |  |
| Amino acid and oligopeptide SLC transporters | Reactome | 84.00 | 7.42 x 10^-16^ | 84.00 | 0.0019 |  |  | 84.00 | 2.50 x 10^-5^ |
| Amino Acid conjugation | Reactome |  |  |  |  | 93.33 | 6.64 x 10^-3^ |  |  |
| Amino acid synthesis and interconversion (transamination) | Reactome | 89.13 | 7.44 x 10^-3^ |  |  | 89.13 | 4.20 x 10^-4^ |  |  |
| Amino acid transport across the plasma membrane | Reactome | 87.50 | 2.21 x 10^-18^ | 87.50 | 8.25 x 10^-4^ | 87.50 | 5.82 x 10^-26^ | 87.50 | 7.19 x 10^-6^ |
| Arginine and proline metabolism | KEGG | 70.51 | 0.0167 |  |  |  |  |  |  |
| Arginine biosynthesis | KEGG | 82.61 | 0.0149 |  |  | 82.61 | 0.0121 |  |  |
| Beta-Alanine metabolism | KEGG |  |  |  |  |  |  | 87.50 | 0.0458 |
| Biogenic amines are oxidatively deaminated to aldehydes | Reactome | 100.00 | 5.03 x 10^-3^ |  |  |  |  |  |  |
| Biosynthesis of maresin conjugates in tissue regeneration | Reactome | 50.00 | 0.0394 |  |  | 50.00 | 0.0355 |  |  |
| Biotin metabolism | KEGG |  |  | 32.14 | 0.0149 |  |  |  |  |
| Biotin transport and metabolism | Reactome |  |  | 88.89 | 0.0133 |  |  |  |  |
| Glutathione conjugation | Reactome |  |  | 87.10 | 0.0442 |  |  |  |  |
| Glutathione metabolism | KEGG |  |  | 57.89 | 0.0361 |  |  |  |  |
| Glutathione synthesis and recycling | Reactome |  |  | 92.86 | 0.0215 |  |  |  |  |
| Branched-chain amino acid catabolism | Reactome | 89.36 | 6.98 x 10^-4^ |  |  | 89.36 | 4.61 x 10^-4^ |  |  |
| Central carbon metabolism in cancer | KEGG | 91.89 | 3.97 x 10^-19^ |  |  | 91.89 | 2.82 x 10^-24^ | 91.89 | 1.31 x 10^-5^ |
| Conjugation of carboxylic acids | Reactome |  |  |  |  | 93.33 | 6.64 x 10^-3^ |  |  |
| Creatine metabolism | Reactome |  |  |  |  | 93.33 | 6.64 x 10^-3^ |  |  |
| CREB phosphorylation through the activation of CaMKII | Reactome | 100.00 | 0.049 |  |  | 100.00 | 0.0442 |  |  |
| Cysteine and methionine metabolism | KEGG | 83.61 | 1.46 x 10^-3^ |  |  | 83.61 | 7.07 x 10^-5^ |  |  |
| Ether lipid metabolism | KEGG | 8.00 | 0.0199 |  |  |  |  |  |  |
| FOXO signaling pathway | KEGG | 80.00 | 0.0394 |  |  |  |  |  |  |
| Glycine serine and threonine metabolism | KEGG |  |  |  |  | 78.00 | 3.45 x 10^-3^ |  |  |
| Glyoxylate and dicarboxylate metabolism | KEGG |  |  |  |  | 72.58 | 7.17 x 10^-3^ |  |  |
| Histidine catabolism | Reactome | 95.65 | 0.0197 |  |  |  |  | 95.65 | 0.0361 |
| Histidine metabolism | KEGG | 68.09 | 0.0398 |  |  |  |  |  |  |
| Histidine, lysine, phenylalanine, tyrosine, proline and tryptophan catabolism | Reactome | 87.91 | 8.71 x 10^-11^ | 87.91 | 0.0067 | 87.91 | 2.22 x 10^-11^ | 87.91 | 2.30 x 10^-6^ |
| Lysine catabolism | Reactome | 78.13 | 0.0251 | 78.13 | 0.0410 | 78.13 | 0.0206 |  |  |
| Lysine degradation | KEGG | 61.82 | 0.0445 |  |  | 61.82 | 2.01 x 10^-4^ |  |  |
| Metabolism of amino acids and derivatives | Reactome | 80.00 | 7.04 x 10^-9^ | 80.00 | 0.0493 | 80 | 7.95 x 10^-11^ | 80.00 | 1.53 x 10^-4^ |
| Metabolism of folate and pterines | Reactome | 72.00 | 0.0134 |  |  |  |  |  |  |
| Metabolism of proteins | Reactome | 48.70 | 1.28 x 10^-9^ |  |  |  |  |  |  |
| Metabolism of vitamins and cofactors | Reactome | 70.00 | 1.77 x 10^-4^ | 70.00 | 6.80 x 10^-4^ | 70.00 | 6.96 x 10^-4^ |  |  |
| Metabolism of water-soluble vitamins and cofactors | Reactome | 72.13 | 1.81 x 10^-4^ | 72.13 | 0.0080 | 72.13 | 9.38 x 10^-4^ |  |  |
| Mineral absorption | KEGG |  |  |  |  |  |  | 89.66 | 0.0426 |
| mTOR signaling pathway | KEGG | 66.67 | 0.0199 |  |  | 66.67 | 0.0179 |  |  |
| Pantothenate and CoA biosynthesis | KEGG | 64.29 | 0.0134 |  |  | 64.29 | 0.0109 |  |  |
| Phenylalanine and tyrosine catabolism | Reactome | 96.77 | 1.86 x 10^-4^ | 96.77 | 0.0490 | 96.77 | 2.24 x 10^-3^ | 96.77 | 8.90 x 10^-6^ |
| Phenylalanine metabolism | KEGG |  |  |  |  |  |  | 76.39 | 0.0032 |
| Phenylalanine tyrosine and tryptophan biosynthesis | KEGG | 68.57 | 1.58 x 10^-3^ | 68.57 | 0.0394 | 68.57 | 1.16 x 10^-3^ | 68.57 | 6.04 x 10^-4^ |
| Post-translational protein modification | Reactome |  |  |  |  | 51.70 | 0.0467 |  |  |
| Protein digestion and absorption | KEGG |  |  | 95.74 | 0.0021 | 95.74 | 4.36 x 10^-21^ | 95.74 | 3.09 x 10^-5^ |
| Proton-coupled neutral amino acid transporters | Reactome |  |  |  |  | 75.00 | 6.50 x 10^-7^ |  |  |
| Purine ribonucleoside monophosphate biosynthesis | Reactome |  |  |  |  | 89.19 | 0.0347 |  |  |
| Ras activation upon Ca2+ influx through NMDA receptor | Reactome | 100.00 | 0.049 |  |  | 100.00 | 0.0442 |  |  |
| SLC-mediated transmembrane transport | Reactome |  |  |  |  | 91.57 | 6.94 x 10^-15^ |  |  |
| Tetrahydrobiopterin (BH4) synthesis, recycling, salvage and regulation | Reactome |  |  | 77.78 | 0.0345 |  |  | 77.78 | 0.0345 |
| Transport of bile salts and organic acids, metal ions and amine compounds | Reactome |  |  |  |  |  |  | 89.74 | 1.18 x 10^-4^ |
| Transport of inorganic cations/anions and amino acids/oligopeptides | Reactome |  |  | 85.71 | 0.0024 | 85.71 | 1.57 x 10^-21^ | 85.71 | 3.76 x 10^-5^ |
| Tryptophan catabolism | Reactome | 88.24 | 4.97 x 10^-14^ | 88.24 | 0.0490 |  |  | 88.24 | 8.90 x 10^-6^ |
| Tryptophan metabolism | Reactome | 62.96 | 1.46 x 10^-3^ |  |  | 88.24 | 7.10 x 10^-11^ |  |  |
| Tyrosine metabolism | KEGG | 80.77 | 0.0239 |  |  | 80.77 | 0.018 |  |  |
| Urea cycle | Reactome | 95.65 | 0.0197 |  |  |  |  |  |  |
| Valine leucine and isoleucine biosynthesis | KEGG | 56.52 | 2.41 x 10^-4^ |  |  | 56.52 | 3.52 x 10^-6^ |  |  |
| Valine leucine and isoleucine degradation | KEGG | 61.90 | 2.01E-03 |  |  | 61.90 | 1.47 x 10^-3^ |  |  |
| Vitamin B6 metabolism | KEGG |  |  | 57.14 | 0.0264 |  |  |  |  |
| Vitamins B6 activation to pyridoxal phosphate | Reactome |  |  | 88.89 | 0.0264 |  |  |  |  |
| Vitamin digestion and absorption | KEGG | 74.36 | 0.0332 |  |  |  |  |  |  |
| ^a^%, percentage of metabolites present in the pathway. | | | | | | | | | |
